# Effectiveness and Success Factors of Bilateral Arm Training After Stroke: A Systematic Review and Meta-Analysis

**DOI:** 10.3389/fnagi.2022.875794

**Published:** 2022-04-25

**Authors:** Siyun Chen, Yuqi Qiu, Clare C. Bassile, Anita Lee, Ruifeng Chen, Dongsheng Xu

**Affiliations:** ^1^College of Rehabilitation Science, Shanghai University of Traditional Chinese Medicine, Shanghai, China; ^2^Engineering Research Center of Traditional Chinese Medicine Intelligent Rehabilitation, Ministry of Education, Shanghai, China; ^3^Department of Rehabilitation and Regenerative Medicine, Columbia University Vagelos College of Physicians and Surgeons, New York, NY, United States; ^4^School of Statistics, East China Normal University, Shanghai, China; ^5^Division of Biostatistics and Bioinformatics, University of California, San Diego, La Jolla, CA, United States

**Keywords:** bilateral arm training, stroke, upper extremity, rehabilitation, ICF model, meta-analysis, neuroplasticity

## Abstract

Bilateral arm training (BAT) presents as a promising approach in upper extremity (UE) rehabilitation after a stroke as it may facilitate neuroplasticity. However, the effectiveness of BAT is inconclusive, and no systematic reviews and meta-analyses have investigated the impact of different factors on the outcomes of BAT. This systematic review and meta-analysis aimed to (1) compare the effects of bilateral arm training (BAT) with unilateral arm training (UAT) and conventional therapy (CT) on the upper limb (UL) motor impairments and functional performance post-stroke, and (2) investigate the different contributing factors that may influence the success of BAT. A comprehensive literature search was performed in five databases. Randomized control trials (RCTs) that met inclusion criteria were selected and assessed for methodological qualities. Data relating to outcome measures, characteristics of participants (stroke chronicity and severity), and features of intervention (type of BAT and dose) were extracted for meta-analysis. With 25 RCTs meeting the inclusion criteria, BAT demonstrated significantly greater improvements in motor impairments as measured by Fugl-Meyer Assessment of Upper Extremity (FMA-UE) than CT (*MD* = 3.94, *p* = < 0.001), but not in functional performance as measured by the pooled outcomes of Action Research Arm Test (ARAT), Box and Block Test (BBT), and the time component of Motor Function Test (WMFT-time) (*SMD* = 0.28, *p* = 0.313). The superior motor impairment effects of BAT were associated with recruiting mildly impaired individuals in the chronic phase of stroke (*MD* = 6.71, *p* < 0.001), and applying a higher dose of intervention (*MD* = 6.52, *p* < 0.001). Subgroup analysis showed that bilateral functional task training (BFTT) improves both motor impairments (*MD* = 7.84, *p* < 0.001) and functional performance (*SMD* = 1.02, *p* = 0.049). No significant differences were detected between BAT and UAT for motor impairment (*MD* = −0.90, *p* = 0.681) or functional performance (*SMD* = −0.09, *p* = 0.457). Thus, our meta-analysis indicates that BAT may be more beneficial than CT in addressing post-stroke UL motor impairment, particularly in the chronic phase with mild UL paresis. The success of BAT may be dose-dependent, and higher doses of intervention may be required. BFTT appears to be a valuable form of BAT that could be integrated into stroke rehabilitation programs. BAT and UAT are generally equivalent in improving UL motor impairments and functional performance.

## Introduction

Contralateral hemiparesis is one of the most common deficits following a stroke (Cramer et al., 1997; Van Der Lee et al., 2001). It is estimated that 48–77% of stroke patients encounter contralateral hemiparesis acutely (Lawrence et al., 2001; Held et al., 2019; Simpson et al., 2021), and 40–50% of patients will continue to have it chronically (Jørgensen et al., 1995; Cramer et al., 1997; Broeks et al., 1999). Although the most significant amount of recovery is suggested to happen in the first three months post-stroke (Wade et al., 1983; Kwakkel et al., 2003; Kwakkel and Kollen, 2013), research has supported that upper extremity (UE) recovery can still occur years after (Carey et al., 1993).

Due to the prevalence of UE impairments post-stroke and the importance of recovery for optimal function and performance of activities of daily living (ADLs), different rehabilitation strategies have been identified and studied. It is not surprising that most of the well-studied UE rehabilitation strategies to date are primarily focused on the unilateral arm since hemiparesis is more evident on one side of the body following a stroke (Beer et al., 2000; Wagner et al., 2006; Sathian et al., 2011; Kantak et al., 2017). However, there is evidence that stroke patients have reduced bilateral arm coordination and functional performance in most ADLs compared to the neurologically intact population (Kantak et al., 2017). In fact, most manual tasks in our daily life require the usage of both UEs and interlimb coordination. A relatively recent upper extremity (UE) rehabilitation strategy targeting interlimb coordination post-stroke is bilateral arm training (BAT). It involves incorporating both upper limbs to perform motor tasks simultaneously or sequentially to improve the movement of the affected limb (Mudie and Matyas, 2000; Waller et al., 2008). Several types of BAT have been identified, including bilateral functional task training (BFTT), bilateral arm training with rhythmic auditory cueing (BATRAC), bilateral robot-assisted training (BRAT), bilateral priming, and mirror therapy (Stoykov and Corcos, 2009; Wolf et al., 2014). Several hypotheses have been proposed regarding the positive effects of BAT on motor function. First, BAT may promote positive neural interactions between sensorimotor-related areas in the ipsilesional and contralesional hemispheres to enhance coupling effects post-stroke (Fan et al., 2015, 2016). Second, increased activity in the sensorimotor-related areas following BAT may contribute to functional reorganization and neuroplasticity (Whitall et al., 2011; Waller et al., 2014). Third, BAT may allow restoration of normalized interhemispheric transcallosal inhibition (IHI) and reduce short-interval intracortical inhibition (SICI) in the ipsilesional hemisphere, both of which are associated with recovery of motor function after stroke (Cicinelli et al., 2003; Stinear et al., 2008; Swayne et al., 2008).

Despite the potential for the usage of BAT in the post-stroke population, the effectiveness of BAT is inconsistent across the studies. According to the Guidelines for Adult Stroke Rehabilitation and Recovery for Healthcare Professionals, bilateral training paradigms fall into Class-IIb in which benefits outweigh the risks, but usefulness/efficacy is less well-established, and additional research is needed (Winstein et al., 2016). The level of evidence is graded as “A” with multiple populations evaluated and data derived from multiple RCTs and meta-analyses. A recent meta-analysis (Chen et al., 2019) compared the effect of BAT with unilateral arm training (UAT) in the post-stroke population based upon the World Health Organization (WHO) International Classification of Functioning, Disability and Health (ICF) framework (World Health Organization, 2001). The results revealed that BAT yielded greater improvements in UE motor impairments but not functional performance. Similar findings have also been reported in other systematic reviews (Stewart et al., 2006; Cauraugh et al., 2010; Latimer et al., 2010; Wolf et al., 2014). However, the results should be cautiously interpreted since these reviews included non-RCTs, and some of the included studies did not comprise a comparison group, rendering it difficult to draw robust conclusions. In contrast, other reviews identified contradictory findings, claiming that BAT was similar or inferior to conventional therapy (CT) or UAT (Coupar et al., 2010; Van Delden et al., 2012; Lee et al., 2017b; Richardson et al., 2021). A recently published systematic review highlighted that UAT and BAT improved paretic UE function equivocally in adults with chronic stroke. Van Delden et al. (2012) also reported similar findings based on the categorization of the ICF framework. However, the small number of studies included in these two reviews may limit their generalizability.

Few studies to date have systematically investigated the factors influencing the success of BAT. Van Delden et al. (2012) pointed out that intervention success may depend on the severity of hemiparesis and time of intervention post-stroke. Additionally, (modified) constraint-induced movement therapy [(m)CIMT] has been reported to be more effective than BAT in one systematic review (Lee et al., 2017b). However, considering that (m)CIMT usually involves patients who are mildly impaired or in a later stage of stroke, it is reasonable to assume that the characteristics of participants are important factors when selecting an optimal intervention. Furthermore, different features of treatment within BAT, such as type and dosage of BAT intervention, may also affect the outcomes (Cooke et al., 2010; Pollock et al., 2014; Wolf et al., 2014). Cauraugh et al. (2010) found that BATRAC and coupled BAT with active stimulation are most effective, whereas Wolf et al. (2014) reported no differences between BFTT, BATRAC, and BRAT. Although the effect of dose of a post-stroke UE treatment has been studied (Kwakkel et al., 1997; Van Peppen et al., 2004; Cooke et al., 2010), no meta-analyses have yet reported the impact of dose on the outcomes of BAT. Therefore, a meta-analysis including high-quality RCTs is urgently needed to systematically investigate the factors influencing the effect of BAT.

Therefore, the purposes of the current systematic review and meta-analysis were twofold: Firstly, to compare the effects of BAT with other interventions in post-stroke UE rehabilitation on motor impairments and functional performance which are two domains of the WHO ICF framework, and secondly, to investigate different determinant factors and their contributions in optimizing comprehensive post-stroke interventions.

## Methods

### Literature and Search Strategy

The Preferred Reporting Items for Systematic reviews and Meta-analyses (PRISMA) was followed for the present review (Page et al., 2021). A computer-based search of the literature was conducted from the date of inception to June 2021 in the following databases: PubMed, MEDLINE, EMBASE, Cochrane, and Web of Science. The databases were searched using a combination of controlled vocabulary (MeSH) and free-text terms related to the patient type “stroke,” body part “upper extremity,” intervention type “bilateral arm training,” and study type “randomized controlled trial” (see Appendix 1). The search strategy was formulated in MEDLINE and modified to the other databases.

### Study Selection

The inclusion criteria to identify for qualifying articles were (a) available in English; (b) randomized control trials (RCTs) that included post-stroke adult participants (over the age of 18 years); (c) reported at least one standardized outcome measure; (d) post-stroke duration of the recruited participants was specified; (e) the intervention used for the experimental group was some form of bilateral arm training (BAT); (f) the control group included either unilateral training, conventional rehabilitation, or both. The exclusion criteria were (a) failure to provide relevant data on the outcome measures; (b) the BAT was used not only in one group; (c) the upper limb intervention was not the only focus of the study (i.e. use of virtual reality or electrostimulation in adjunction to UL intervention). Titles and abstracts were screened by two reviewers independently and compared against pre-determined eligibility criteria. All relevant studies were then reviewed in full text to confirm if the inclusion criteria were fulfilled. If any discrepancies arose, a third reviewer was consulted and made the final decision.

### Quality Assessment

Physiotherapy Evidence Database (PEDro) scale was used to assess the methodological quality of each included RCT by two independent reviewers. The PEDro scale is an 11-item scale that has been widely used for rating the methodological quality of RCTs (Sherrington et al., 2000; Maher et al., 2003). Each satisfied item (item 2–11) pertained to internal validity was given one point to the total PEDro scale (maximum score = 10 points), and item 1 related to external validity was rated a YES or NO. Studies scoring four or higher on the PEDro scale were considered of sufficient quality (Van Peppen et al., 2004). Included RCTs were rated by two independent reviewers, and a third reviewer made the final decision if any discrepancies occurred.

### Data Collection

The following information was extracted independently by two reviewers from the included studies: (1) baseline characteristics of the study participants (age, gender, side of lesion, post-stroke duration); (2) interventions implemented in experimental and control groups (type, duration, and intensity); (3) the inclusion and exclusion criteria; (4) The outcome measures data; (5) whether the study includes follow-up data and the timeline for data collection. Based upon the WHO ICF framework (World Health Organization, 2001), the outcome measures included in this review primarily focused on two domains: (1) motor impairment associated with body functions and structure and (2) functional performance of upper limb (UL) associated with activities. Fugl-Meyer Assessment of Upper Extremity (FMA-UE) data was collected to represent body functions and structure domain, and Action Research Arm Test (ARAT), Box and Block Test (BBT), and the time component of Motor Function Test (WMFT-time) were extracted to represent activities domain since they measure the same underlying construct (Coupar et al., 2010). Motor Activity Log (MAL) is a self-reported assessment of patients' perspective of arm use and quality of movement in ADLs (Santisteban et al., 2016). The MAL was also extracted to represent patient-perceived UL functional performance if available. If more than one outcome measure was reported for the same domain in the identified article, only one outcome measure's data was extracted. WMFT was pooled first if more than one outcome measures were reported for the ICF activities domain, followed by ARAT, BBT, and MAL. The order was determined based on the reported frequency of use and the level of measurement quality and clinical utility (Alt Murphy et al., 2015; Santisteban et al., 2016). The reviewers reached a consensus through discussion or consulting the third reviewer if disagreement occurred.

**Figure 1:**
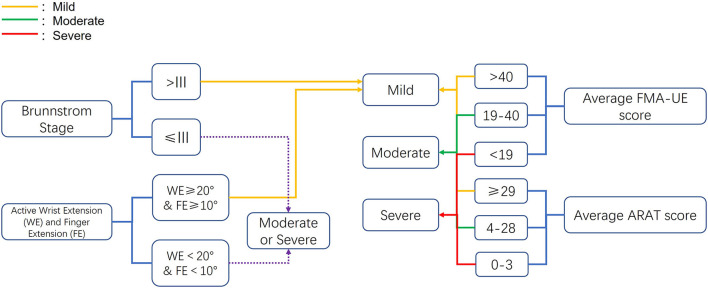
Criteria for the upper extremity paresis severity classification. ARAT, Action Research Arm Test; FMA-UE, Fugl-Meyer Assessment of Upper Extremity.

To compare the effects of BAT with CT and UAT according to the severity of UL paresis, we adopted and revised the severity classification based upon the previous studies (Van Der Lee et al., 1999; Suputtitada et al., 2004; Morris et al., 2008; Lin et al., 2009; Stoykov et al., 2009; Wu et al., 2011) included in Van Delden and colleagues' work (Van Delden et al., 2012). As shown in Figure 1, the hemiparesis severity classification was based on Brunnstrom stage, active range of motion of wrist and finger extension, average baseline FMA-UE score, and ARAT score. The recruited participants in each study were categorized into mild, moderate, and severe UL paresis.

Since another focus of this review was to explore the effects of bilateral training during different phases of stroke in comparison to other rehabilitation protocols, we classified the included studies into three recovery stages based on the timeline of stroke recovery established in the first Stroke Recovery and Rehabilitation Roundtable (Bernhardt et al., 2017): hyper-acute/acute (0–7 days post-stroke), subacute (7 days−6 months post-stroke) and chronic (>6 months post-stroke).

The type of interventions implemented in the experimental and comparison groups were documented for subgroup analysis. Bilateral arm training involves performing motor tasks with both ULs in a symmetric or asymmetric design. It can be practiced with or without the aid of an external device (Hatem et al., 2016). As described in a previous review, three categories of BAT, including bilateral functional task training (BFTT), bilateral arm training with rhythmic auditory cueing (BATRAC), and bilateral robot-assisted training (BRAT) have been identified (Wolf et al., 2014). In addition to the three categories of BAT mentioned above, mirror therapy (MT) involves the use of a mirror to create a reflective illusion of the non-affected arm as if it were the affected one (Ramachandran et al., 1995). One strategy of MT involves actively synchronizing the affected limb with the mirror reflection of the unaffected limb, thus considering it as a form of BAT (Toh and Fong, 2012). Most of the previous systematic reviews examining the effectiveness of BAT excluded MT (Van Delden et al., 2012; Chen et al., 2019; Richardson et al., 2021). However, MT is cost-friendly, simple, and relatively less labor-intensive, and its effectiveness is of interest to be examined with other types of BAT. Therefore, we included the active form of MT as a type of BAT in the present review. In the case of BRAT and BFTT, if BFTT were used simultaneously in the same experimental therapy protocol, it was considered BRAT since an external robotic device was used.

For the control subgroups, unilateral arm training (UAT) was described as an exercise intervention using the hemiparetic UL while excluding the contralateral UL (Van Delden et al., 2012) as well as conventional therapy (CT), which involved conventional occupational/physical therapy, routine clinical rehabilitation, traditional therapeutic activities, dose-matched therapeutic exercise that did not exclude the use of the non-paretic arm were identified.

Data related to the intervention dose was also extracted to conduct a subgroup analysis. Therapy dose can be described in terms of length of treatment sessions, the number of treatment sessions, and intensity of intervention (Cooke et al., 2010; Pollock et al., 2014). The intervention dose often provides duration-based information in stroke rehabilitation, including minutes or days per week (Lang et al., 2009). Therefore, we determined a criterion to categorize the included studies into higher and lower dose groups. If the treatment hours were ≥7 hours per week or the total treatment hours ≥ 30, it was classified into the higher dose group. Whereas if the treatment hours were <7 h per week or the total treatment hours <30 h, it was categorized into the lower dose group. For the studies reporting the range of training time, the average value was used to calculate the intervention dose.

For the studies with more than 2 groups (e.g., contains BAT, UAT and CT groups), BAT vs. UAT and BAT vs. CT data were extracted separately for meta-analyses. All the extracted data were summarized in the tables.

### Statistical Analysis

Data management and meta-analysis were performed by R version 3.6.3 using “metafor” package (Viechtbauer, 2010). Mean difference (MD) and standardized mean difference [SMD, assessed by Hedges' g, *g* = 0.2 for small effect, *g* = 0.5 for medium effect, and *g* = 0.8 for large effect (Cohen, 1988)] between post and pre-intervention was used as the primary outcome for motor impairment measurement and the function performance measurements, respectively. For studies that only reported mean and standard deviation for post and pre-intervention rather than the MD, we calculated the MD or SMD as appropriate. We obtained the sample standard deviation (SD) for MD or SMD using the covariance formula with correlation parameter ρ = 0.8 (Chen et al., 2019; Deeks et al., 2021). Finally, the estimated MD or SMD and the corresponding 95% confidence interval (CI) were reported. The results were visualized using forest plots.

The MD of FMA-UE was used to test motor impairment, while the SMD of ARAT, BBT, and WMFT-time were aggregated for testing function performance. Although MAL is also on the UL functional performance level of the WHO ICF, it represents patient-perceived UL activity performance and was hence analyzed separately as a secondary analysis. SMD was calculated for MAL for easier comparison with other functional outcomes. Two null hypotheses were tested for both motor impairment and functional performance in the meta-analysis: (1). There is no difference in MD or SMD between BAT and CT; and (2). There is no difference in MD or SMD between BAT and UAT. Mixed-effects model was used for the meta-analysis regression. The Higgins *I*^2^ index and Q-statistic were used to test the heterogeneity. The τ^2^ (estimated amount of total heterogeneity) and *H*^2^ (total variability divided by sampling variability) were also estimated and reported. A fixed-effects model would be used if *I*^2^ < 50%, indicating a low-to-moderate heterogeneity; otherwise, a random-effects model would be used.

Several subgroup analyses were performed. MD of motor impairment and SMD of function performance were investigated by stroke chronicity (three phases), the severity of UL paresis (three phases), the type of BAT (four types), and the intervention dose (two doses).

Publication bias was evaluated by funnel plot virtually and then tested by rank-correlation test and Egger's test (Begg and Mazumdar, 1994; Egger et al., 1997). For analysis that may have publication bias, the selection models method with step function was performed and tested by likelihood ratio test (LRT) as the sensitivity analysis (Hedges, 1992; Hedges and Vevea, 1996; Mcshane et al., 2016).

## Results

### Data Retrieval

**Figure 2:**
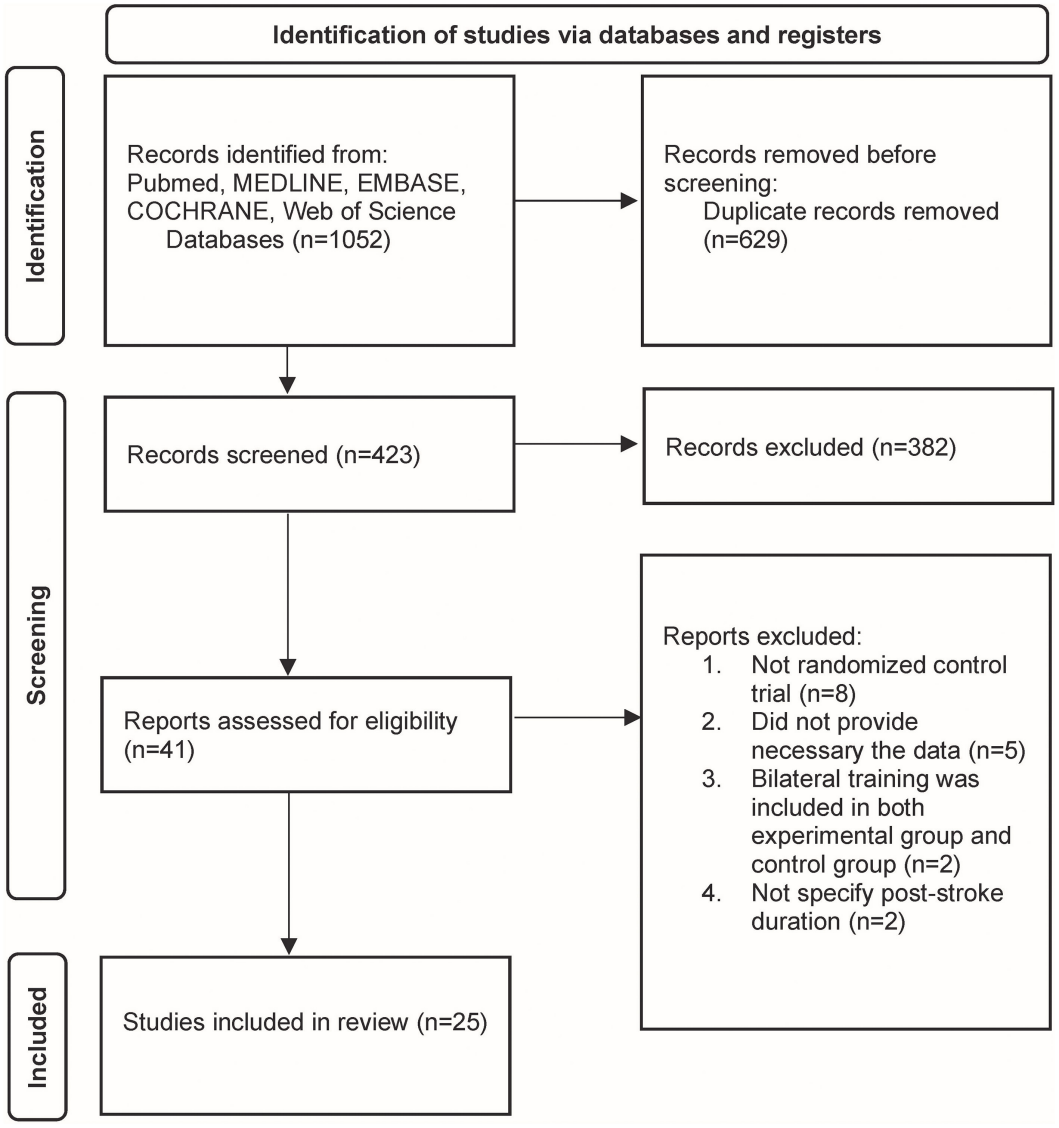
The Preferred Reporting Items for Systematic Reviews and Meta-Analyses (PRISMA) flowchart of study identification.

The initial search resulted in 1,052 articles. Once duplicate articles were excluded, 423 articles remained for further screening. Therefore, 382 articles were removed subsequently based on title and abstract. Forty-one articles were subjected to full-text assessment, of which 17 articles were excluded due to the following reasons: (i) the studies were not RCTs (*n* = 8), (ii) the studies did not provide sufficient data for statistical analysis (*n* = 5), (iii) the bilateral training was included in both experimental and control groups (*n* = 2), (iv) post-stroke duration was not specified in the study (*n* = 2). Finally, a total of 25 studies with 1,103 participants fulfilled the inclusion criteria and were ultimately included in this systematic review (Figure 2).

### Study Characteristics

One study included participants in the acute phase of stroke (Meng et al., 2018). Eight studies recruited subjects in the subacute phase of stroke (Desrosiers et al., 2005; Lum et al., 2006; Morris et al., 2008; Brunner et al., 2012; Van Delden et al., 2013; Samuelkamaleshkumar et al., 2014; Hsieh et al., 2017; Renner et al., 2020), and the remaining 16 studies included subjects in the chronic phase of stroke (Luft et al., 2004; Waller and Whitall, 2008; Lin et al., 2009, 2010, 2015; Hsieh et al., 2011; Whitall et al., 2011; Wu et al., 2011, 2013a,b; Liao et al., 2012; Yang et al., 2012; Lee et al., 2017a; Sethy et al., 2018; Hsu et al., 2019; Hung et al., 2019).

Based upon the pre-determined criteria for the severity of UL paresis, two studies classified as targeting populations with severe paresis (Samuelkamaleshkumar et al., 2014; Renner et al., 2020), nine studies were considered as including participants with a moderate paresis (Luft et al., 2004; Lum et al., 2006; Morris et al., 2008; Waller and Whitall, 2008; Whitall et al., 2011; Liao et al., 2012; Hsieh et al., 2017; Meng et al., 2018; Hung et al., 2019), and participants recruited in the remaining 14 studies were categorized as having mild UL paresis (Desrosiers et al., 2005; Lin et al., 2009, 2010, 2015; Hsieh et al., 2011; Wu et al., 2011, 2013a,b; Brunner et al., 2012; Yang et al., 2012; Van Delden et al., 2013; Lee et al., 2017a; Sethy et al., 2018; Hsu et al., 2019).

Four categories of BAT were identified in the included studies: (1) bilateral functional task training (BFTT) (*n* = 10) (Desrosiers et al., 2005; Morris et al., 2008; Lin et al., 2009, 2010; Wu et al., 2011; Brunner et al., 2012; Lee et al., 2017a; Meng et al., 2018; Sethy et al., 2018; Renner et al., 2020), (2) bilateral robot-assisted training (BRAT) (*n* = 9) (Lum et al., 2006; Hsieh et al., 2011, 2017; Liao et al., 2012; Yang et al., 2012; Wu et al., 2013b; Lin et al., 2015; Hsu et al., 2019; Hung et al., 2019), (3) mirror therapy (MT) (*n* = 2) (Wu et al., 2013a; Samuelkamaleshkumar et al., 2014), and (4) bilateral training with rhythmic auditory cueing (BATRAC) (*n* = 4) (Luft et al., 2004; Waller and Whitall, 2008; Whitall et al., 2011; Van Delden et al., 2013). One study implemented a hybrid therapy protocol (participants received an equal amount of time in BFTT and BRAT in the experimental groups) was classified into the BRAT group since an external robotic device was used (Hung et al., 2019).

Two studies were excluded from the subgroup analysis for the intervention dose due to the unequal amount of therapy received in the experimental and control groups (Brunner et al., 2012; Samuelkamaleshkumar et al., 2014), yielding 23 total studies available for further investigation. According to the previously described criteria, 13 studies were classified into the higher dose group (Lin et al., 2009, 2010; Hsieh et al., 2011, 2017; Wu et al., 2011, 2013a,b; Liao et al., 2012; Yang et al., 2012; Lee et al., 2017a; Meng et al., 2018; Sethy et al., 2018; Renner et al., 2020), while 10 were categorized into the lower dose group (Luft et al., 2004; Desrosiers et al., 2005; Lum et al., 2006; Morris et al., 2008; Waller and Whitall, 2008; Whitall et al., 2011; Van Delden et al., 2013; Lin et al., 2015; Hsu et al., 2019; Hung et al., 2019).

The rehabilitation protocols used in the control groups included conventional therapy (CT) (*n* = 21) and unilateral arm training (UAT) (*n* = 10). The reported protocols of UAT consisted of (modified) constraint-induced movement therapy (m)CIMT (*n* = 2) (Brunner et al., 2012; Van Delden et al., 2013) distributed constraint-induced therapy (dCIT) (*n* = 2) (Lin et al., 2009; Wu et al., 2011) unilateral robotic-assisted therapy (URAT) (*n* = 4), (Lum et al., 2006; Yang et al., 2012; Wu et al., 2013b; Hung et al., 2019), and dose-matched unilateral functional task training (UFTT) (*n* = 2) (Morris et al., 2008; Renner et al., 2020). Twenty-one studies had conventional therapy as a comparison group that included multidisciplinary rehabilitation programs, sensorimotor stimulation program, task-oriented approach, routine occupational therapy, or physical therapy.

The baseline information of participants and the characteristics of included studies are present in Tables 1, 2.

**Table 1:** Characteristics of recruited participants included in this meta-analysis.

**References**	**Total N**	**Age (years) [Mean (SD)]**	**Gender [Male (%)]**	**Time post stroke [Mean (SD)]**	**Lesion side [Right (%)]**	**Phase**	**Severity**
Brunner et al. (2012)	30	BAT = 64.8 (12.8); UAT = 61.0 (10.0)	BAT = 8 (50.0%); UAT = 11 (78.6%)	BAT = 36.9 d (25.1); UAT = 48.43 d (39.3)	BAT = 10 (62.5%); UAT = 8 (57.1%)	Subacute	Mild
Desrosiers et al. (2005)	41	BAT = 72.2 (10.8); CT = 74.3 (10.1)	BAT = 9 (45.0%); CT = 10 (47.6%)	BAT = 34.2 d (34.4); CT = 35.4 d (33.7)	BAT = 7 (35.0%); CT = 11 (52.4%)	Subacute	Mild
Hsieh et al. (2011)	18	BAT = 54.6 (10.5); CT = 54.0 (8.05)	BAT = 8 (66.7%); CT = 5 (83.3%)	BAT = 18.0 mo (8.0); CT = 28.33 mo (19.9)	BAT = 6 (50.0%); CT = 2 (33.3%)	Chronic	Mild
Hsieh et al. (2017)	31	BAT = 49.28 (10.9); CT = 52.87 (10.40)	BAT = 11 (68.8%); CT = 7 (46.7%)	BAT = 2.5 mo (1.69); CT = 2.21 mo (1.11)	BAT = 8 (50.0%); CT = 11 (73.3%)	Subacute	Moderate
Hsu et al. (2019)	43	BAT = 53.1 (13.9); CT = 52.6(12.5)	BAT = 11 (50.0%); CT = 9 (42.9%)	BAT = 13.7 mo (8.6); CT = 14.7 mo (13.2)	NR	Chronic	Mild
Hung et al. (2019)	29	BAT = 58.45 (13.11); UAT = 53.17 (12.28)	BAT = 10 (66.7%); UAT = 9 (64.3%)	BAT = 29.33 mo (28.44); UAT = 37.86 mo (34.77)	BAT = 6 (40.0%); UAT = 8 (57.1%)	Chronic	Moderate
Lee et al. (2017a)	30	BAT = 57.33 (9.88); CT = 54.60 (16.03)	BAT = 9 (60.0%); CT = 10 (66.7%)	NR	BAT = 7 (46.7%); CT = 6 (40.0%)	Chronic	Mild
Liao et al. (2012)	20	BAT = 55.51 (11.17); CT = 54.56 (8.20)	BAT = 6 (60.0%); CT = 7 (70.0%)	BAT = 23.9 mo (13.39); CT = 22.20 mo (17.47)	BAT = 6 (60.0%); CT = 7 (70.0%)	Chronic	Moderate
Lin et al. (2009)	60	BAT = 51.58 (8.67); UAT = 55.28 (9.34); CT = 50.70 (13.93)	BAT = 12 (60.0%); UAT = 11 (55.0%); CT = 11 (55.0%)	BAT = 18.50 mo (17.40); UAT = 21.25 mo (21.59); CT = 21.90 mo (20.51)	BAT = 11 (55.0%); UAT = 8 (40.0%); CT = 12 (60.0%)	Chronic	Mild
Lin et al. (2010)	33	BAT = 52.08 (9.60); CT = 55.50 (13.17)	BAT = 10 (62.5%); CT = 9 (52.9%)	BAT = 13.94 mo (12.73); CT = 13.12 mo (8.13)	BAT = 7 (43.8%); CT = 9 (52.9%)	Chronic	Mild
Lin et al. (2015)	33	BAT = 52.63 (10.49); CT = 57.47 (10.29)	BAT = 12 (75.0%); CT = 16 (94.1%)	BAT = 27.75 mo (19.04); CT = 21.82 mo (21.66)	BAT = 8 (50.0%); CT = 8 (47.1%)	Chronic	Mild
Luft et al. (2004)	21	BAT = 63.5 (15.3); CT = 59.6 (10.5)	BAT = 7 (77.8%); CT = 4 (36.4%)	*BAT = 75 mo (NR); *CT = 45.5 mo (NR)	BAT = 6 (66.7%); CT = 7 (63.6%)	Chronic	Moderate
Lum et al. (2006)	20	BAT = 72.2 (11.7); UAT = 69.8 (4.0); CT = 59.9 (5.5)	BAT = 2 (40.0%); UAT = 5 (55.6%); CT = 4 (66.7%)	BAT = 6.2 wk (1.0); UAT = 10.0 wk (1.9); CT = 10.6 wk (2.7)	BAT = 3 (60.0%); UAT = 5 (55.6%); CT = 4 (66.7%)	Subacute	Moderate
Meng et al. (2018)	128	BAT = 55.38 (6.97); CT = 55.19 (7.82)	BAT = 34 (53.1%); CT = 31 (48.4%)	BAT = 8.87 hr (2.69); CT = 9.08 hr (2.35)	BAT = 35 (54.7%); CT = 33 (51.6%)	Acute	Moderate
Morris et al. (2008)	106	BAT = 67.9 (13.1); UAT = 67.8 (9.9)	BAT = 34 (60.7%); UAT = 27 (54.0%)	BAT = 22.6 d (5.6); UAT = 23.2 d (5.7)	BAT = 29 (51.8%); UAT = 23 (46.0%)	Subacute	Moderate
Renner et al. (2020)	69	BAT = 63.7(12.39); UAT = 63.3 (12.50)	BAT = 16 (45.7%); UAT = 16 (47.1%)	BAT = 35.2d(11.03); UAT = 37.2d (13.6)	BAT = 19 (54.3%); UAT = 22 (64.7%)	Subacute	Severe
Samuelkamaleshkumar et al. (2014)	20	BAT = 48.4 (15.58); CT = 53.9 (11.57)	BAT = 8 (80.0%); CT = 8 (80.0%)	BAT = 3.7 wk (1.1); CT = 4.4 wk (1.4)	BAT = 6 (60.0%); CT = 4 (40.0%)	Subacute	Severe
Sethy et al. (2018)	28	BAT = 57.34 (11.92); CT = 57.59 (11.03)	BAT = 10 (71.4%); CT = 9 (64.3%)	BAT = 13.09 mo (2.86); CT = 13.82 mo(3.01)	BAT = 5 (35.7%); CT = 4 (28.6%)	Chronic	Mild
Van Delden et al. (2013)	60	BAT = 62.6 (9.8); UAT = 59.8 (13.8); CT = 56.9 (12.7)	BAT = 11 (57.9%); UAT = 14 (63.6%); CT = 16 (84.2%)	BAT = 7.8 wk (4.9); UAT = 9.2 wk (6.8); CT = 11.1 wk (6.8)	BAT = 11 (57.9%); UAT = 12 (54.5%); CT = 11 (57.9%)	Subacute	Moderate
Waller and Whitall (2008)	18	BAT = 57.95 (13.11); CT = 54.06 (9.11)	BAT = 5 (55.6%); CT = 2 (22.2%)	BAT = 73.53 mo (73.97); CT = 31.55 mo (23.52)	BAT = 4 (44.4%); CT = 4 (44.4%)	Chronic	Moderate
Whitall et al. (2011)	92	BAT = 59.8 (9.9); CT = 57.7 (12.5)	BAT = 26 (61.9%); CT = 24 (48.0%)	BAT = 4.5 yr (4.1); CT = 4.1 yr (5.2)	BT = 23 (56.1%); CT = 25 (50.0%)	Chronic	Moderate
Wu et al. (2011)	66	BAT = 52.22 (10.72); UAT = 51.91 (11.93); CT = 55.19 (2.50)	BAT = 18 (81.8%); UAT = 15 (68.2%); CT = 16 (72.7%)	BAT = 15.92 mo (13.74); UAT = 14.91 mo (12.41); CT = 17.77 mo (12.45)	BAT = 12 (54.5%); UAT = 8 (36.4%); CT = 10 (45.5%)	Chronic	Mild
Wu et al. (2013a)	33	BAT = 54.77 (11.66); CT = 53.59 (10.21)	BAT = 11 (68.8%); CT = 12 (70.6%)	BAT = 19.31 mo (12.57); CT = 21.88 mo (15.55)	BAT = 8 (50.0%); CT = 10 (58.8%)	Chronic	Mild
Wu et al. (2013a)	53	BAT = 52.21 (12.20); UAT = 54.95 (9.90); CT = 54.22 (9.78)	BAT = 13 (72.2%); UAT = 10 (55.6%); CT = 12 (70.6%)	BAT = 23.28 mo (15.37); UAT = 19.00 mo (15.51); CT = 23.41 mo (15.24)	BAT = 9 (50.0%); UAT = 12 (66.7%); CT = 8 (47.1%)	Chronic	Mild
Yang et al. (2012)	21	BAT = 51.4 (10.9); UAT = 50.8 (6.1); CT = 51.6 (7.6)	BAT = 4 (57.1%); UAT = 5 (71.4%); CT = 5 (71.4%)	BAT = 14.7 mo (5.7); UAT = 12.3 mo (4.4); CT = 14.3 mo (6.8)	BAT = 4 (57.1%); UAT = 4 (57.1%); CT = 3 (42.9%)	Chronic	Mild

*Data are presented as median; BAT, bilateral arm training; CT, conventional therapy; d, days; hr, hours; mo, months; NR, not reported; SD, standard deviation; UAT, unilateral arm training; wk, weeks; yr, years.

**Table 2:** Characteristics of the included studies in this meta-analysis.

**References**	**Type of BAT**	**Comparison group**	**BAT intervention dose**	**Outcome measures**
Brunner et al. (2012)	BFTT	UAT: (m)CIMT	4 hr/wk with an OT/PT, daily self-training exercise 2–3 hours/d, 4 wk Total: 86 hrs	ARAT, 9HPT, MAL
Desrosiers et al. (2005)	BFTT	CT: functional activities and exercises	45 min/session, 4 d/wk, 15–20 total sessions, 5 wk Total: 13.125 hrs	FMA, vigorimeter, BBT, Purdue Pegboard Test, FTNT, TEMPA, FIM, AMPS
Hsieh et al. (2011)	BRAT	CT: conventional OT	90–105 min/session, 5 d/wk, 4 wk Total: 32.5 hrs	FMA, MRC scale, MAL, the ABILHAND scale, urinary 8-OHdG, MFSI
Hsieh et al. (2017)	BRAT	CT: task-oriented approach followed by functional and real-life tasks	90 min/session, 5 d/wk, 4 wk Total: 30 hrs	FMA, dynamometer, BBT, modified Rankin Scale, FIM, actigraphy, SIS, self-reported fatigue scale
Hsu et al. (2019)	BRAT	CT: sensorimotor stimulation program followed by therapist-facilitated task-specific training	50 min/session 3 d/wk, 4 wk Total: 10 hrs	MAL,FMA,sEMG
Hung et al. (2019)	BRAT + BFTT	UAT: URAT + (m)CIMT	90 min/session, 3 d/wk, 6 wk Total: 27 hrs	FMA, SIS, WMFT, NEADL
Lee et al. (2017a)	BFTT	CT: OT incorporated the Bobath approach	60 min/session, 5 d/wk, 8 wk Total: 40 hrs	FMA, BBT, MBI
Liao et al. (2012)	BRAT	CT: OT training	90-105 min/session, 5 d/wk, 4wk Total: 32.5 hrs	arm activity ratio, FMA, FIM, MAL, ABILHAND questionnaire
Lin et al. (2009)	BFTT	UAT: dCIT CT: conventional exercises	120 min/session, 5 d/wk, 3 wk Total: 30 hrs	FMA, FIM, MAL, SIS
Lin et al. (2010)	BFTT	CT: OT incorporated NDT	120 min/session, 5 d/wk, 3 wk Total: 30 hrs	Kinematic analyses, FMA, FIM, MAL
Lin et al. (2015)	BRAT	CT: routine clinical rehabilitation	30 min/session, 3 d/wk, 4 wk Total: 6 hrs	BI, FMA, MAS, WMFT
Luft et al. (2004)	BATRAC	CT: based on NDT principles	60 min/session, 3 d/wk, 6 wk Total: 18 hrs	FMA, WMAT, UMAQS, dynamometry, fMRI
Lum et al. (2006)	BRAT	UAT: UFTT CT: conventional therapy based on NDT	60 min/session, 15 total sessions, 4 wk Total: 15 hrs	modified Ashworth scale, FMA, FIM, MSS, motor power examination
Meng et al. (2018)	BFTT	CT: conventional rehabilitation program	60 min/session, 2 session/d, 5 d/wk, 2 wk Total: 20 hrs	FMA, ARAT, AMP, RMT, CMCT
Morris et al. (2008)	BFTT	UAT: UFTT	20 min/session, 5 d/wk, 6 wk Total: 10 hrs	ARAT, RMA UL scale, 9HPT, MBI, Hospital Anxiety and Depression Scale, Nottingham Health Profile
Renner et al. (2020)	BFTT	UAT: UFTT	arm cycle: 20 min/session, 2 sessions/d+ progressive BT/UT session: 20 min/session, 1 session/d. 5 d/wk, 6 wk Total: 30 hrs	FMA, biomechanical parameters measuring isometric force and rate of force generation
Samuelkamaleshkumar et al. (2014)	MT	CT: multidisciplinary rehabilitation program	30 min/session, 2 sessions/d, 5 d/wk, 3 wk Total: 15 hrs	FMA, Brunnstrom stages of motor recovery, BBT, modified Ashworth Scale
Sethy et al. (2018)	BFTT	CT: conventional OT based on Bobath approach.	60 min/session, 5 d/wk, 6 wk Total: 30 hrs	FMA, ARAT, MAL
Van Delden et al. (2013)	mBATRAC	UAT: (m)CIMT CT: exercise therapy based on Royal Dutch Society of Physical Therapy and Dutch Society of Occupational Therapy	60 min/session, 3 d/wk, 6 wk Total: 18 hrs	ARAT, MI, FMA, 9HPT, Erasmus modifications of the Nottingham Sensory Assessment, MAL, SIS
Waller and Whitall (2008)	BATRAC	CT: based on NDT principles	60 min/session, 3 d/wk, 6 wk Total:18 hrs	FMA, WMFT
Whitall et al. (2011)	BATRAC	CT: based on NDT principles	60 min/session, 3 d/wk, 6wk Total:18 hrs	FMA, WMFT, SIS, dynamometer, ROM, 5-point Likert scale, fMRI
Wu et al. (2011)	BFTT	UAT: dCIIT CT: based on NDT principles	120 min/session, 5 d/wk, 3 wk Total: 30 hrs	kinematic variables, WMFT, MAL
Wu et al. (2013a)	MT	CT: traditional therapeutic activities base on task-oriented treatment principles	90 min/session, 3 d/wk, 4 wk Total: 30 hrs	FMA, kinematic variables, the Revised Nottingham Sensory Assessment, MAL, the ABILHAND questionnaire
Wu et al. (2013b)	BRAT	UAT: URAT CT: conventional therapeutic activities	90–105 min/session, 5 d/wk, 4 wk Total: 32.5 hrs	Kinematic variables, WMFT, MAL, ABILHAND Questionnaire
Yang et al. (2012)	BRAT	UAT: URAT CT: conventional therapeutic activities	90–105 min/session, 5 d/wk, 4 wk Total: 32.5 hrs	FMA, MRC instrument, grip strength, Modified Ashworth Scale

*AMAT, Arm Motor Ability Test; AMP, motor-evoked potential amplitude; AMPS, Assessment of Motor and Process Skills; ARAT, Action Research Arm Test; BAT, bilateral arm training; BATRAC, bilateral training with rhythmic auditory cueing; BBT, Box & Block Test; BFTT, bilateral functional task training; BI, The Barthel Index; BRAT, bilateral robot-assisted training; CAHAI-9, Chedoke Arm & Hand Activity Index-9; CMCT, central motor conduction time; COPM, Canadian Occupational Performance Measure; CT, conventional therapy; UAT, unilateral arm training; dCIT, distributed constraint-induced therapy; FIM, Functional Independence Measure; fMRI, Functional magnetic resonance imaging; FTNT, finger to nose test; MAL, Motor Activity Log; (m)CIMT, (modified) constrain-induced movement therapy; MAS, Motor Assessment Score; mBATRAC, modified bilateral training with rhythmic auditory cueing MBI, modified Barthel Index; MFSI, Multidimensional Fatigue Symptom Inventory; MI, Motricity Index; MRC, Medical Research Council; MSS, Motor Status Scale; NDT, Neurodevelopmental Treatment; NEADL, Nottingham Extended Activities of Daily Living; NIHSS, National Institutes of Health Stroke Scale; OT, Occupational Therapy; RMA UL scale, Rivermead Motor Assessment upper-limb scale; RMT, resting motion threshold; sEMG, surface Electromyography; TEMPA, Test d'Evaluation des Membres Supe'rieurs de Personnes Age'es; TMS, Transcranial Magnetic Stimulation; UFTT, unilateral functional task training; UMAQS, University of Maryland Arm Questionnaire for Stroke; URAT, unilateral robot-assisted therapy; SIS, Stroke Impact Scale; WMFT, Wolf Motor Function Test; Y-BAT, Yonsei-Bilateral Activity Test; 9HPT, Nine-Hole Peg Test*.

### Quality Assessment

Table 3 presents the methodological quality assessment of the included studies as evaluated using the PEDro scale. All studies scored more than 4 points on the PEDdro scale, indicating sufficient quality among the included studies. The mean score of PEDro was 6.36 points (*SD* = 0.91), ranging from 5 to 8 points.

**Table 3:** Methodological quality of included studies assessed by Physiotherapy Evidence Database (PEDro) scale.

**References**	**Eligibility criteria specified (Yes/No)**	**Random allocation**	**Concealed allocation**	**Comparable at baseline**	**Blind subjects**	**Blind therapists**	**Blind assessors**	**Adequate follow-up**	**Intention-to-treat analysis**	**Between group comparisons**	**Point estimates and variability**	**PEDro total score (0–10)**
Brunner et al. (2012)	Yes	1	1	1	0	0	1	1	0	1	1	7
Desrosiers et al. (2005)	Yes	1	1	1	0	0	1	1	0	1	1	7
Hsieh et al. (2011)	Yes	1	1	1	0	0	1	1	1	1	1	8
Hsieh et al. (2017)	Yes	1	1	1	0	0	1	1	0	1	1	7
Hsu et al. (2019)	Yes	1	1	1	0	0	1	1	0	1	1	7
Hung et al. (2019)	Yes	1	0	0	0	0	1	1	1	1	1	6
Lee et al. (2017a)	Yes	1	0	1	0	0	1	1	0	1	1	6
Liao et al. (2012)	Yes	1	1	1	0	0	1	1	0	1	1	7
Lin et al. (2009)	Yes	1	1	1	0	0	1	1	0	1	1	7
Lin et al. (2010)	Yes	1	0	0	0	0	1	1	0	1	1	5
Lin et al. (2015)	Yes	1	0	1	0	0	1	1	0	1	1	6
Luft et al. (2004)	Yes	1	0	1	0	0	1	0	0	1	1	5
Lum et al. (2006)	Yes	1	0	0	0	0	1	1	0	1	1	5
Meng et al. (2018)	Yes	1	1	1	0	0	1	1	0	1	1	7
Morris et al. (2008)	Yes	1	1	1	0	0	1	1	1	1	1	8
Renner et al. (2020)	Yes	1	0	1	0	0	1	0	0	1	1	5
Samuelkamaleshkumar et al. (2014)	Yes	1	0	1	0	0	1	1	0	1	1	6
Sethy et al. (2018)	Yes	1	1	1	0	0	1	1	0	1	1	7
Van Delden et al. (2013)	Yes	1	1	1	0	0	1	1	0	1	1	7
Waller and Whitall (2008)	Yes	1	0	0	0	0	1	1	0	1	1	5
Whitall et al. (2011)	Yes	1	0	1	0	0	1	0	1	1	1	6
Wu et al. (2011)	Yes	1	0	1	0	0	1	1	0	1	1	6
Wu et al. (2013a)	Yes	1	1	1	0	0	1	0	0	1	1	6
Wu et al. (2013b)	Yes	1	0	1	0	0	1	1	1	1	1	7
Yang et al. (2012)	Yes	1	0	1	0	0	1	1	0	1	1	6

### Meta-Analysis Results

#### BAT vs. CT

**Figure 3:**
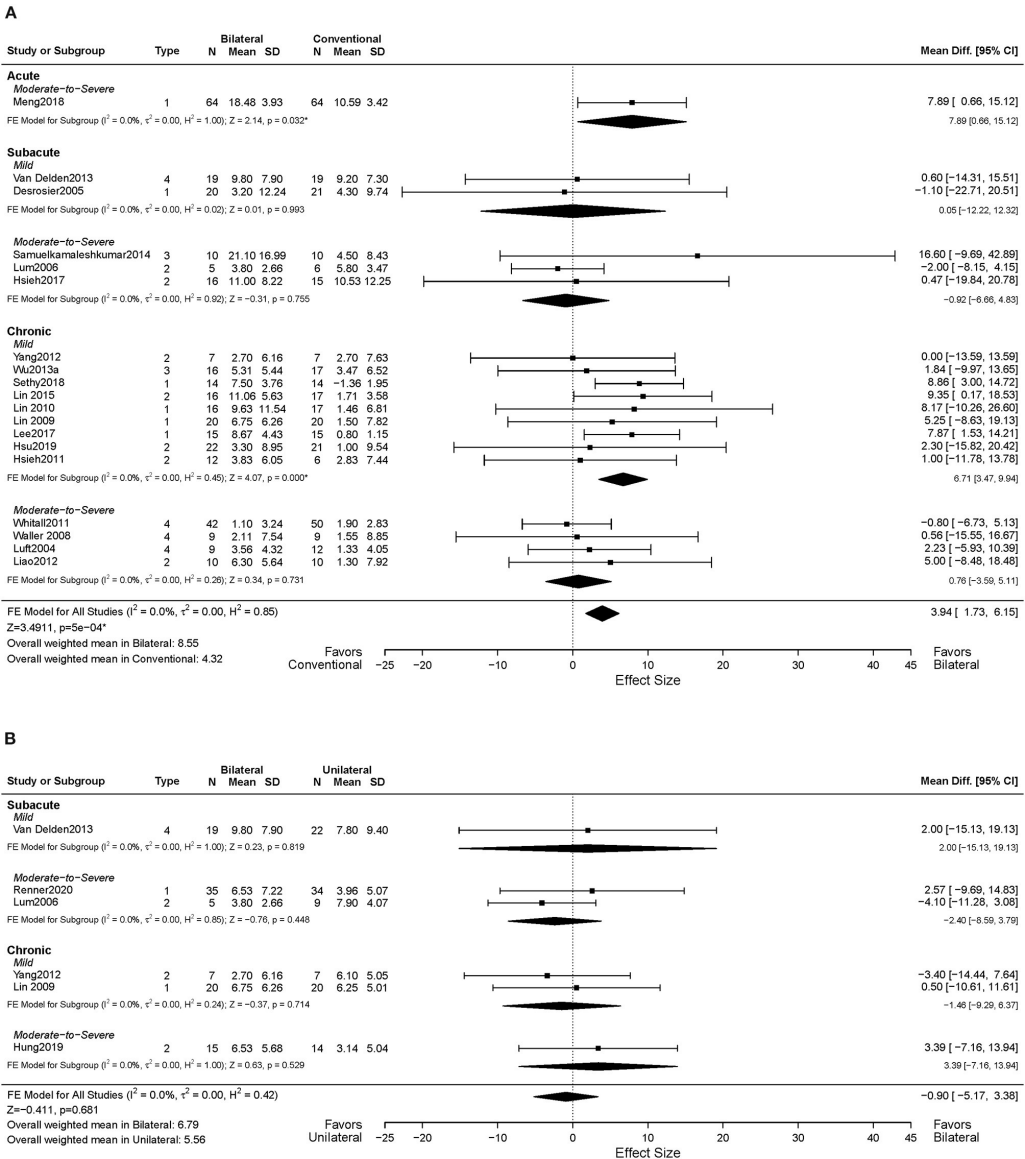
Forest plots comparing the effects of **(A)** BAT vs. CT and **(B)** BAT vs. UAT on the upper extremity motor impairment. 1 = bilateral functional training Test (BFTT); 2 = bilateral robot-assisted training (BRAT); 3 = bilateral arm training with rhythmic auditory cueing (BATRAC); 4 = mirror therapy (MT); BAT, bilateral arm training; CI, confidence interval; CT, conventional therapy; FE, fixed-effects; RE, random-effects; Std. Mean Diff., standardized mean difference; UAT, unilateral arm training; * indicates statistically significant (*p* < 0.05).

Nineteen studies assessed motor impairment of the UL using the FMA-UE (Luft et al., 2004; Desrosiers et al., 2005; Lum et al., 2006; Waller and Whitall, 2008; Lin et al., 2009, 2010, 2015; Hsieh et al., 2011, 2017; Whitall et al., 2011; Liao et al., 2012; Yang et al., 2012; Van Delden et al., 2013; Wu et al., 2013a; Samuelkamaleshkumar et al., 2014; Lee et al., 2017a; Meng et al., 2018; Sethy et al., 2018; Hsu et al., 2019). Figure 3A shows the descriptive and meta-regression results for the motor impairment between BAT and CT. The meta-analysis fixed-effects model found that BAT demonstrated significantly greater UE motor impairments improvements than the CT group (*MD* = 3.94, 95% CI: [1.73, 6.15], *p* < 0.001).

In terms of the time post-stroke, 13 out of the 19 studies recruited participants in the chronic phase of stroke (Luft et al., 2004; Waller and Whitall, 2008; Lin et al., 2010, 2015; Hsieh et al., 2011; Whitall et al., 2011; Liao et al., 2012; Yang et al., 2012; Wu et al., 2013a; Lee et al., 2017a; Sethy et al., 2018; Hsu et al., 2019), and the remaining six studies included subjects in the acute and subacute phases of stroke (Desrosiers et al., 2005; Lum et al., 2006; Van Delden et al., 2013; Samuelkamaleshkumar et al., 2014; Hsieh et al., 2017; Meng et al., 2018). Since only one article was identified in the acute phase, only subacute and chronic phases were considered here. Greater improvements were shown in the chronic phase of stroke with the BAT in FMA-UE compared to the CT (*MD* = 4.59, 95% CI: [2.00, 7.19], *p* < 0.001). However, such differences were not observed in the subacute phase (*MD* = −0.74, 95% CI: [−5.94, 4.46], *p* = 0.780).

Eleven of 19 studies included subjects with mild UL paresis (Lin et al., 2009, 2010, 2015; Hsieh et al., 2011; Yang et al., 2012; Van Delden et al., 2013; Wu et al., 2013a; Lee et al., 2017a; Sethy et al., 2018; Hsu et al., 2019), and eight studies recruited subjects with moderate to severe UL paresis (Luft et al., 2004; Lum et al., 2006; Waller and Whitall, 2008; Whitall et al., 2011; Liao et al., 2012; Samuelkamaleshkumar et al., 2014; Hsieh et al., 2017; Meng et al., 2018). Compared with CT, we found a significant MD in favor of BAT with the mild UL paresis (*MD* = 6.28, 95% CI: [3.15, 9.40], *p* < 0.001), but this effect was not detected in participants with moderate to severe UL paresis (*MD* = 1.60, 95% CI: [−1.53, 4.73], *p* = 0.316). Furthermore, as shown in Figure 3A, the effect size increased if patients were in the chronic phase with the mild UL paresis (*MD* = 6.71, 95% CI: [3.47, 9.94], *p* < 0.001), which is 4.27 times the *MD* = 1.57 of the CT group with chronic phase and mild UL paresis.

The impacts of BAT types on UL motor impairment post-stroke were also investigated. Six of 19 studies applied BFTT (Desrosiers et al., 2005; Lin et al., 2009, 2010; Lee et al., 2017b; Meng et al., 2018; Sethy et al., 2018), BRAT was used in seven studies (Lum et al., 2006; Hsieh et al., 2011, 2017; Liao et al., 2012; Yang et al., 2012; Lin et al., 2015; Hsu et al., 2019), MT was implemented in two studies (Wu et al., 2013a; Samuelkamaleshkumar et al., 2014), and the remaining four studies used (m)BATRAC as a type of BAT (Luft et al., 2004; Waller and Whitall, 2008; Whitall et al., 2011; Van Delden et al., 2013). Only the BFTT group demonstrated significantly greater gains in UL motor impairment than the CT group (*MD* = 7.84, 95% CI: [4.37, 11.30], *p* < 0.001) (Supplementary Figure 1A), whereas the other types of BAT did not illustrate a superior effect.

**Figure 4:**
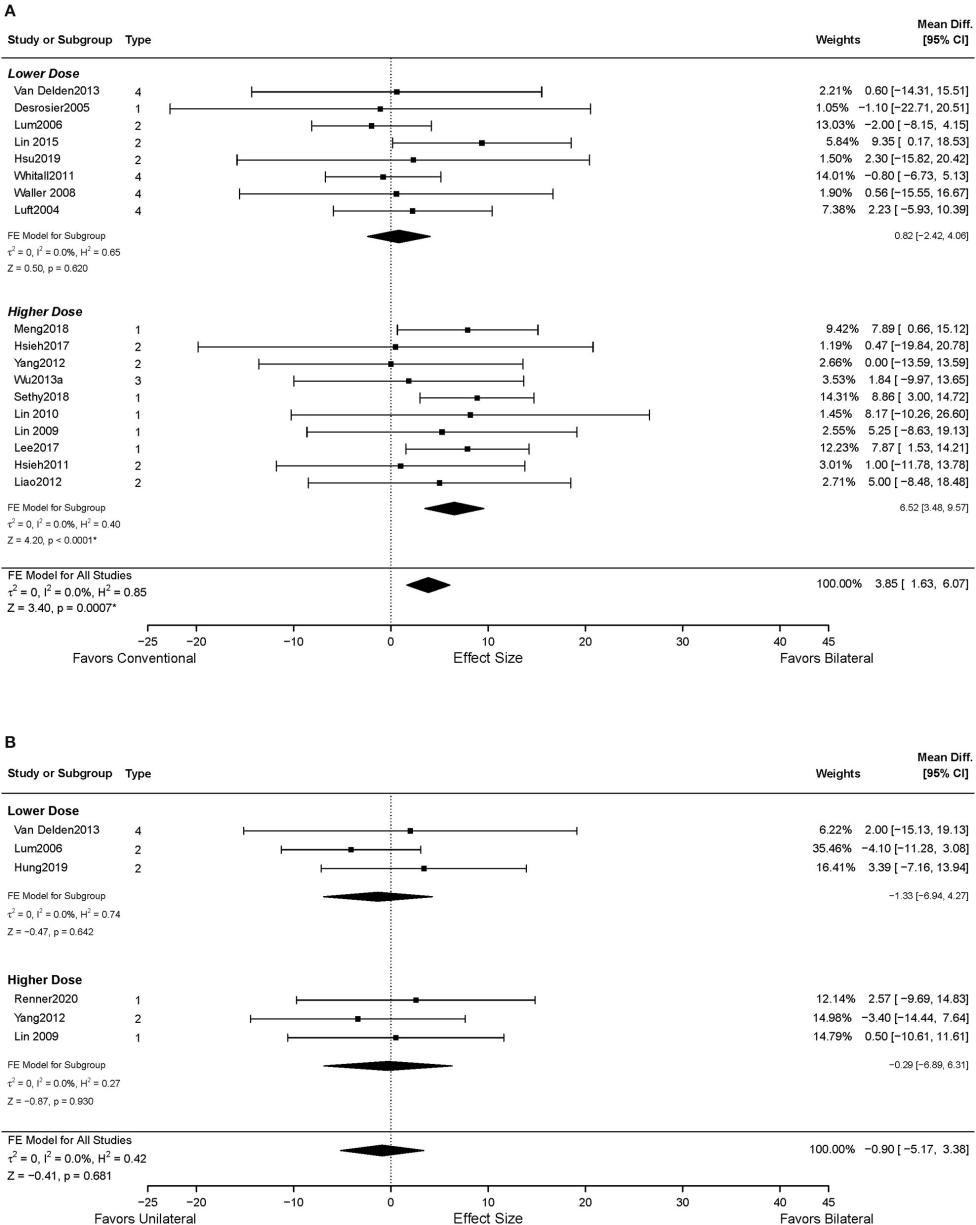
The effects of intervention dose on the UE motor impairment. **(A)** BAT vs. CT; **(B)** BAT vs. UAT. 1 = bilateral functional training Test (BFTT); 2 = bilateral robot-assisted training (BRAT); 3 = bilateral arm training with rhythmic auditory cueing (BATRAC); 4 = mirror therapy (MT); BAT, bilateral arm training; CI, confidence interval; CT, conventional therapy; FE, fixed-effects; RE, random-effects; Std. Mean Diff., standardized mean difference; UAT, unilateral arm training; * indicates statistically significant (*p* < 0.05).

Data from 18 studies assessed FMA-UE were available for the intervention dose subgroup analysis. Ten studies were categorized into the higher dose group (Lin et al., 2009, 2010; Hsieh et al., 2011, 2017; Liao et al., 2012; Yang et al., 2012; Wu et al., 2013a; Lee et al., 2017a; Meng et al., 2018; Sethy et al., 2018), while eight studies were classified into the lower dose group based on the pre-determined criteria (Luft et al., 2004; Desrosiers et al., 2005; Lum et al., 2006; Waller and Whitall, 2008; Whitall et al., 2011; Van Delden et al., 2013; Lin et al., 2015; Hsu et al., 2019). As shown in Figure 4A, significant improvements in motor impairment were observed in the BAT group than the CT group, when the dose of intervention was high (*MD* = 6.52, 95% CI: [3.48, 9.57], *p* < 0.001). However, the differential effect of BAT and CT was not observed with the lower dose training (*MD* = 0.82, 95% CI: [−2.42, 4.06], *p* = 0.620).

**Figure 5:**
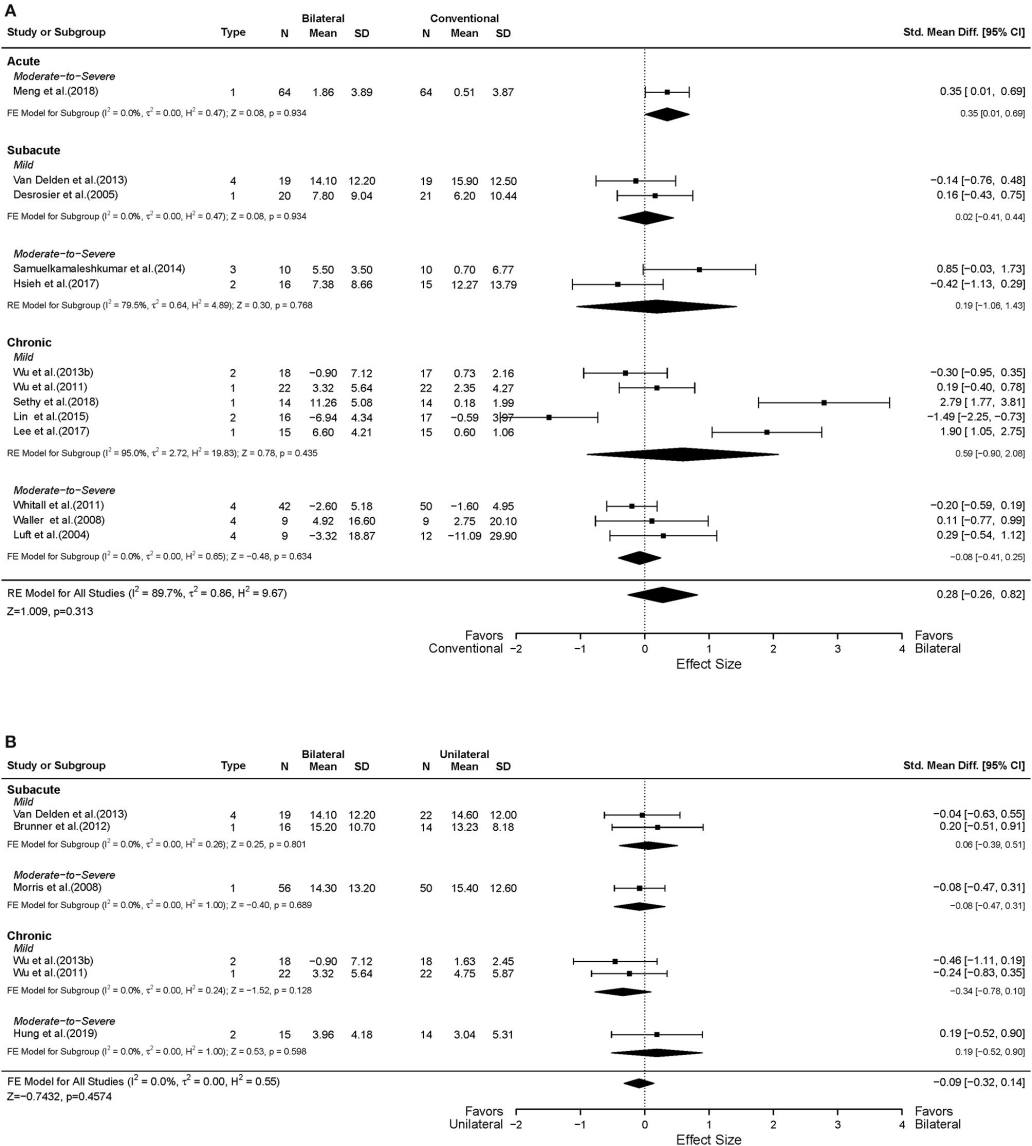
Forest plots comparing the effects of **(A)** BAT vs. CT and **(B)** BAT vs. UAT on the upper extremity functional performance. 1 = bilateral functional training Test (BFTT); 2 = bilateral robot-assisted training (BRAT); 3 = bilateral arm training with rhythmic auditory cueing (BATRAC); 4 = mirror therapy (MT); BAT, bilateral arm training; CI, confidence interval; CT, conventional therapy; FE, fixed-effects; RE, random-effects; Std. Mean Diff., standardized mean difference; UAT, unilateral arm training.

**Figure 6:**
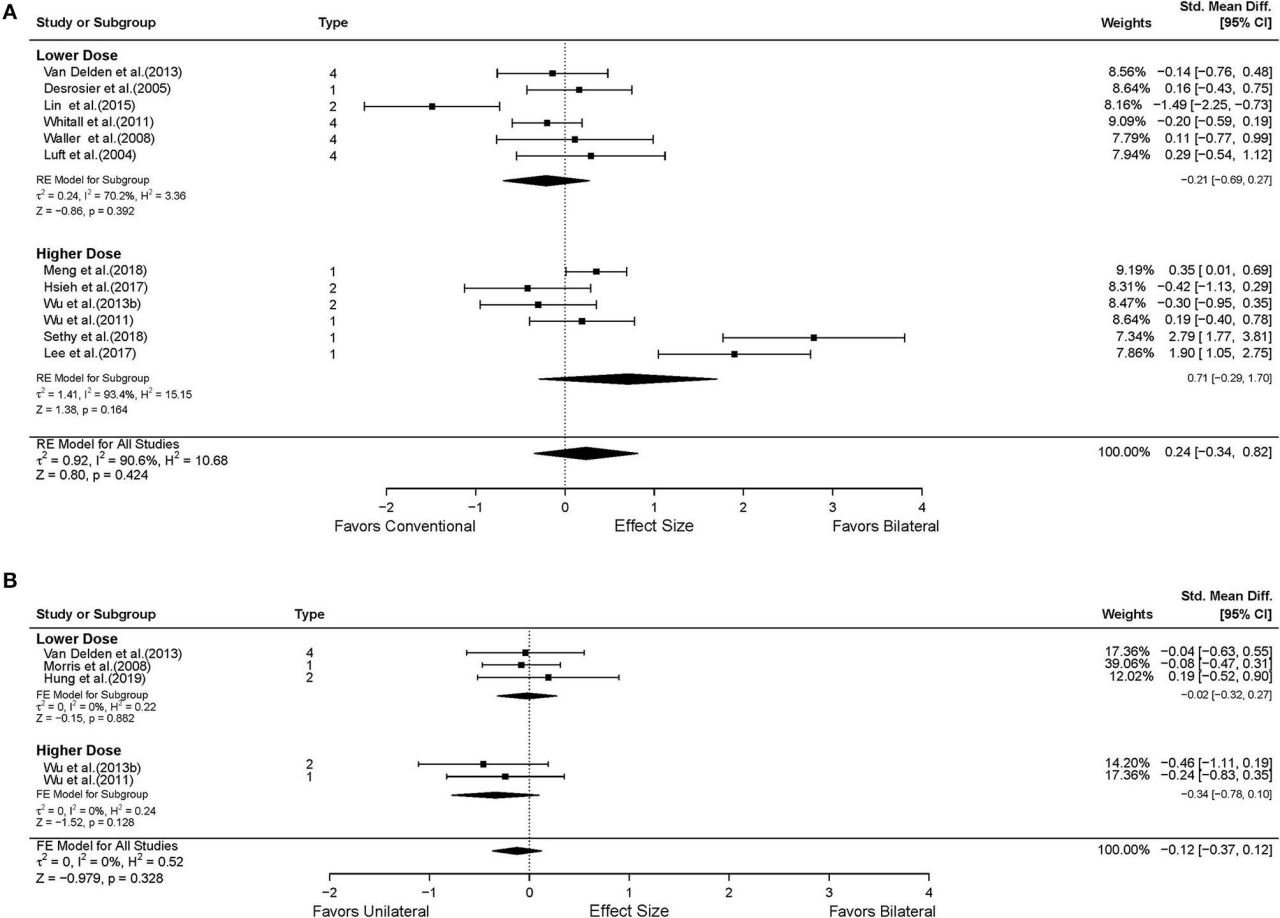
The effects of intervention dose on the UE functional performance. **(A)** BAT vs. CT; **(B)** BAT vs. UAT. 1 = bilateral functional training Test (BFTT); 2 = bilateral robot-assisted training (BRAT); 3 = bilateral arm training with rhythmic auditory cueing (BATRAC); 4 = mirror therapy (MT); BAT, bilateral arm training; CI, confidence interval; CT, conventional therapy; FE, fixed-effects; RE, random-effects; Std. Mean Diff., standardized mean difference; UAT, unilateral arm training.

Thirteen studies reported UL functional performance outcomes using ARAT, BBT, and WMFT-time (Luft et al., 2004; Desrosiers et al., 2005; Waller and Whitall, 2008; Whitall et al., 2011; Wu et al., 2011, 2013b; Van Delden et al., 2013; Samuelkamaleshkumar et al., 2014; Lin et al., 2015; Hsieh et al., 2017; Lee et al., 2017a; Meng et al., 2018; Sethy et al., 2018). MAL data reported from five studies were available for secondary analysis (Lin et al., 2009; Liao et al., 2012; Wu et al., 2013a; Hsu et al., 2019). No differential effect of BAT and CT was found either in the UL functional performance (*SMD* = 0.28, 95% CI: [−0.26, 0.82], *p* = 0.313) (Figure 5A) or in the patient-perceived arm use (*SMD* = 0.02, 95% CI: [−0.28, 0.32], *p* = 0.916) (Supplementary Figure 2A) and quality of movement (*SMD* = 0.08, 95% CI: [−0.22, 0.38], *p* = 0.604) (Supplementary Figure 3A). However, in the subgroup analysis by type of BAT, the results showed that BFTT (*n* = 5) significantly improved UL functional performance as measured by ARAT, BBT and WMFT with a substantially large effect size (*SMD* = 1.02, 95% CI: [0.01, 2.02], *p* = 0.049) (Supplementary Figure 1B). The superior effect was not observed in other types of BAT [*SMD* = −0.72 in BRAT, 0.85 in MT, −0.09 in (m)BATRAC]. No differential effect was noted in the subgroup analysis stratified by intervention dose (Figure 6A).

#### BAT vs. UAT

Six studies in total reported UL motor impairment outcomes using FMA-UE (Lum et al., 2006; Yang et al., 2012; Van Delden et al., 2013; Hung et al., 2019; Renner et al., 2020). No statistically significant difference was found between BAT and UAT in motor impairment (*MD* = −0.90, 95% CI: [−5.17, 3.38], *p* = 0.681) (Figure 3B) or in any subgroup analyses (Figures 3B, 4B).

Six studies assessed the UL functional performance using ARAT, and WMFT-time (Morris et al., 2008; Wu et al., 2011, 2013b; Brunner et al., 2012; Van Delden et al., 2013; Hung et al., 2019). Only one study reported MAL (Lin et al., 2009); the perceived UL functional performance would not be discussed below due to insufficient power (Supplementary Figures 2B, 3B). No significant differences were observed between UAT and BAT regarding the UL functional performance post-stroke in general (*SMD* = −0.09, 95% CI: [−0.32, 0.14], *p* = 0.457) (Figure 5B) or in the subgroup analyses (Figures 5B, 6B).

### Publication Bias and Sensitivity Analyses

**Figure 7:**
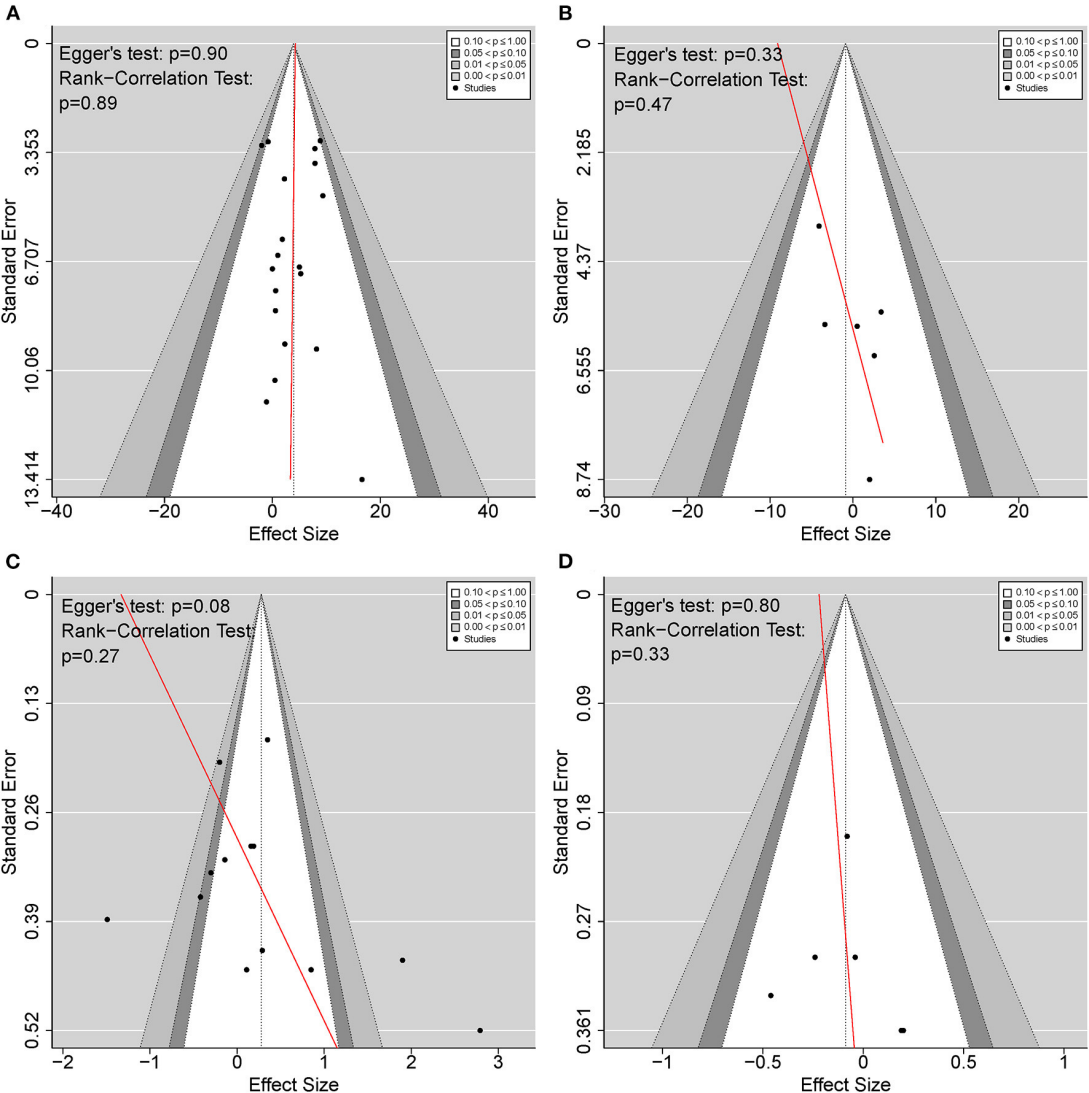
Funnel plots detecting publication bias. **(A)** BAT vs. CT on the upper extremity motor impairment; **(B)** BAT vs. UAT on the upper extremity motor impairment; **(C)** BAT vs. CT on the upper extremity functional performance; **(D)** BAT vs. UAT on the upper extremity functional performance. BAT, bilateral arm training; CT, conventional therapy; UAT, unilateral arm training.

To detect publication bias, Figure 7 shows the funnel plots for BAT compared with CT and UAT, in both motor impairment and functional performance. The red regression line indicates Egger's test for each comparison. There was no publication bias detected as indicated by Egger's test and rank-correlation test.

## Discussion

The purpose of this systematic review and meta-analysis was to evaluate the effectiveness of bilateral arm training (BAT) compared to unilateral arm training (UAT) and conventional therapy (CT), respectively, in changing upper extremity (UE) motor impairments and functional performance in the post-stroke population who have experienced UE hemiparesis. Furthermore, we also aimed to explore different contributing factors in determining optimal intervention post-stroke systematically.

The current meta-analysis revealed that BAT is overall more effective in improving UL motor impairment than CT. However, no differential effects of BAT and CT were observed in terms of enhancing UL functional performance. A review article by Coupar et al. (2010) also reported a favorable effect of BAT in improving motor impairment as measured by FMA-UE based on four studies comparing the effectiveness of bilateral training with usual care. A total of 19 RCTs were included in our current meta-analysis to compare the differential effects of BAT and CT on motor impairment outcome, which greatly improved the robustness of current findings. Additionally, the minimal clinically important difference (MCID) is used as an index to determine whether changes in outcome scores resulting from interventions indicate meaningful and clinically important improvements for patients (Copay et al., 2007; Revicki et al., 2008). The estimated MCID for FMA-UE scores ranged from 4.25 to 7.25 (Page et al., 2012). Our findings showed that while the CT group (*MD* = 4.32) has just reached the lower bound of MCID, the BAT group (*MD* = 8.55) has exceeded the upper bound. The differences between BAT and CT in improving FMA-UE scores were even more substantial in the subgroup analysis.

Few studies to date have examined the effectiveness of different types of BAT in comparison to CT. A previous systematic review based on two studies found that the BFTT group showed greater gains in FMA-UE scores than the CT group but not in the activity measures (Wolf et al., 2014). With more RCTs (*n* = 7), our results were partially consistent with their findings, indicating that implementing BFTT not only resulted in improved motor impairment but also UL functional performance. BFTT typically involves repetitions of various bilateral UL activities with complex interlimb coordination. Repeated attempts to achieve functional task goals associated with BFTT increase ipsilesional hemisphere excitability and help restore balanced IHI (Harris-Love et al., 2011), both of which are associated with better functional recovery post-stroke (Cicinelli et al., 2003; Koski et al., 2004; Murase et al., 2004; Calautti et al., 2007). Moreover, as previously stated in the literature, repetitive practice of functional tasks and asymmetrical movements involves a problem-solving process. It requires greater brain activation in the motor-related cortical areas that may facilitate learning-dependent neuroplasticity (Sadato et al., 1997; Guadagnoli and Lee, 2004; Timmermans et al., 2010; Tazoe et al., 2013). Thus, increased activation in the ipsilesional hemisphere, restoration of normalized IHI, and promotion of learning-based neuroplasticity might explain the improvements in motor impairment and functional performance in BFTT. Interestingly, although functional performance was improved according to laboratory-based outcome measures, no significant differences were reported in patient-perceived functioning as measured by MAL. Previous evidence suggested that patients' perceptions of functional changes reflect rehabilitation outcomes as effectively as laboratory-based functional measures; however, the former may require larger sample sizes to overcome measurement errors (Simpson and Eng, 2013). Thus, the limited studies reporting MAL in the BFTT group (*n* = 2) might explain the differences between patient-perceived functional performance and other pooled functional outcomes in the current meta-analysis. Nevertheless, some practice tasks in the BFTT protocol and test items in the outcome measures are overlapped; the learning effects could not be ruled out in our study; thus, the results should be interpreted with caution. No significant improvements in motor impairments or functional performance were found with BRAT, BATRAC, or MT in this meta-analysis. Conversely, Cauraugh et al. (2010) reported that BATRAC and coupled bilateral training protocols are the most effective in improving motor capabilities. It is noted that most of the studies included in their review were non-RCTs with diverse comparison groups, and the outcome measures utilized to represent motor recovery were not under the same construction based on the ICF framework. Thus, these limitations might contribute to the observed discrepancy with our findings. Unlike BRAT and BATRAC, BFTT is low-cost, does not require the assistance of an external device, and contains more diversity of UL activities. Our findings encourage rehabilitation professionals to incorporate BFTT into their stroke motor recovery clinical practice.

In the subgroup analysis by phase and severity of the stroke, we found that compared to CT, BAT is the most efficacious in improving motor impairment when applied to subjects with mild UL paresis in the chronic phase of stroke. Although motor improvements were shown in the subacute stage, no differences between BAT and CT were detected. Previous research stated that the neurological recovery does not display a linear pattern, with most patients experiencing some degree of spontaneous recovery post-stroke (Kwakkel et al., 2006; Langhorne et al., 2011). Evidence has supported that the majority of spontaneous recovery of motor function occurs in the first three months post-stroke (Wade et al., 1983; Kwakkel et al., 2003; Kwakkel and Kollen, 2013). Thus, in the subacute stages of recovery, motor function gains may not be so closely related to rehabilitative interventions (BAT or CT) but rather to spontaneous neurological recovery, which might explain the observed equivalent effects of BAT and CT. Additionally, among the five studies with the largest effect size, four applied BFTT as a form of BAT intervention. In this phase, relatively high-functioning individuals with stroke usually show persistent deficits in fine motor dexterity, finger strength, and force control (Patel et al., 2020). Most BFTT protocols involve training that requires fine motor dexterity, such as buttoning, reaching and grasping small objects, tracing, etc. As stated above, higher levels of brain activation in the motor-related cortex have been reported when performing fine tasks with asymmetrical movements (Sadato et al., 1997; Tazoe et al., 2013). Therefore, interventions to improve fine motor dexterity to induce a greater brain activation for favorable effects of BAT in clinical practice may require in individuals with mild UL paresis in the chronic phase of stroke.

Additionally, although it is generally supported by previous studies that higher doses of exercise therapy are somewhat associated with improved motor outcome post-stroke (Kwakkel et al., 1997; Van Peppen et al., 2004; Cooke et al., 2010), no meta-analyses to date have investigated the impact of dose of BAT on motor recovery post-stroke. Based on the included 18 RCTs in the subgroup analysis, BAT significantly improved motor impairments compared with dose-matched CT in the higher dose group. However, BAT and CT did not demonstrate any differences in gaining motor function in the lower dose group. Our current finding indicated that the effects of BAT are dose-dependent and that the beneficial effects of BAT may only be seen with doses of intervention after stroke. Therefore, the intervention dose should be carefully considered when incorporating BAT into clinical practice.

Consistent with the findings of previous reviews (Coupar et al., 2010; Van Delden et al., 2012; Hatem et al., 2016; Lee et al., 2017b), our results indicated that BAT and UAT showed equivocal effects either in improving UL motor impairment or functional performance post-stroke in general. However, the present review did not support the reported superior effect of BAT in improving motor impairment post-stroke as indicated by FMA-UE compared to UAT in Chen et al.'s study (Chen et al., 2019). It should be noted that protocols such as conventional training and routine clinical rehabilitation program were categorized into the UAT group in their review, rendering it challenging to make an accurate comparison between BAT and UAT. In fact, most CT and dose-matched therapeutic exercises protocols were based on neurodevelopmental techniques or multidisciplinary rehabilitation programs consisting of compensatory practice or a small component of bilateral functional task training, both of which include using the unaffected arm. Our results also reported different findings when examining the efficacy of BAT in comparison to CT and UAT, respectively. Therefore, distinguishing CT and UAT into two different comparison groups is necessary for future studies to precisely capture the effects of different types of intervention.

Publication bias was not found in the included studies. However, a potential asymmetry appeared in Figure 6C with Egger's test reporting a *p*-value > 0.05 but < 0.1. To be extra cautious, we further investigated the three identified studies that primarily accounted for the asymmetry (Lin et al., 2015; Lee et al., 2017a; Sethy et al., 2018). A common problem across all three studies was the small sample size, insufficient power, and the effect size was either too high or too low, rendering the results less reliable. However, after excluding the above studies, the heterogeneity disappeared, and the sensitivity analysis results were consistent with the current results. Therefore, these three studies did not affect our findings of the current meta-analysis.

### Limitations

The present systematic review and meta-analysis had some limitations. First, we only extracted data related to UL motor impairment and functional performance immediately after the intervention. The long-term efficacy of BAT compared to UAT and CT was not evaluated because the limited studies (*n* = 9) provided follow-up data and the period for follow-up data collection varied largely among these studies. The long-term effectiveness of BAT should be investigated in future studies since it holds significant value in clinical practice. Second, due to largely varied inclusion and exclusion criteria and outcome measures utilized in the included studies, categorizing the severity of UL paresis was difficult. According to our pre-defined criteria, the studies were classified into different severity levels based on the mean values of baseline outcome measures. However, the severity differences within each study may have been overlooked. Third, in contrast to the number of studies included in BAT vs. CT, the number of studies comparing the efficacy of BAT with UAT and subgroup analyses were relatively small, which makes the generalization of the results in BAT vs. UAT less reliable. Fourth, although the use of random-effects model, heterogeneity may still potentially interfere with the interpretation of BAT and CT findings on functional performance after stroke. Fifth, the inclusion criteria were limited to include studies published in English. Not having reviewed the Chinese literature systematically may have potentially missed relevant studies.

## Conclusion

In conclusion, the present systematic review and meta-analysis suggested that BAT might be more beneficial than conventional therapy CT to improve upper limb (UL) motor impairment post-stroke. The current study also highlighted stroke chronicity, the severity of impairment, type of treatment, and intervention dose were critical factors in choosing an optimal rehabilitation program for restoring UE motor function. BAT might be especially more efficacious than CT in addressing motor impairment if a higher dose of intervention was applied or recruited patients in the chronic phase post-stroke had mild UL paresis. It is also suggested that BFTT may be a valuable form of BAT as it may facilitate both motor and functional recovery; therefore, due to its low cost, simplicity, and variety of activities, it is highly recommended to be integrated into stroke rehabilitation programs. BAT and UAT are generally equivalent in improving UL motor impairments and functional performance post-stroke. However, future comparisons based on a larger number of high-quality studies are needed to precisely capture the differential effects of UAT and BAT.

## Data Availability Statement

The raw data supporting the conclusions of this article will be made available by the authors, without undue reservation.

## Author Contributions

SC and YQ conceived the idea for the manuscript and participated in the study design. SC, YQ, and AL participated in the literature search and data acquisition. YQ and RC performed data and statistical analysis. SC, CB, AL, YQ, and DX were involved in manuscript preparation, editing, and review. All authors have read and approved the final version of the manuscript, and agree with the order of presentation of the authors.

## Funding

This work was supported by National Key R&D Program of China [grant number: 2020YFC2004202] and National Natural Science Foundation of China [grant number: 81974358].

## Conflict of Interest

The authors declare that the research was conducted in the absence of any commercial or financial relationships that could be construed as a potential conflict of interest.

## Publisher's Note

All claims expressed in this article are solely those of the authors and do not necessarily represent those of their affiliated organizations, or those of the publisher, the editors and the reviewers. Any product that may be evaluated in this article, or claim that may be made by its manufacturer, is not guaranteed or endorsed by the publisher.

## Supplementary Material

Appendix 1 Search strategies for meta-analysis.

Supplementary Figure 1 The effects of bilateral functional task training (BFTT) on the upper extremity **(A)** motor impairments and **(B)** functional performance. BAT, bilateral arm training; CI, confidence interval; CT, conventional therapy; FE, fixed-effects; RE, random-effects; Std. Mean Diff., standardized mean difference. ^*^indicates statistically significant (*p* < 0.05).

Supplementary Figure 2 Forest plots comparing the effects of **(A)** BAT vs. CT and **(B)** BAT vs. UAT on the MAL-amount of use. 1 = bilateral functional training Test (BFTT); 2 = bilateral robot-assisted training (BRAT); 3 = bilateral arm training with rhythmic auditory cueing (BATRAC); 4 = mirror therapy (MT); BAT, bilateral arm training; CI, confidence interval; CT, conventional therapy; FE, fixed-effects; RE, random-effects; Std. Mean Diff., standardized mean difference; UAT, unilateral arm training; ^*^indicates statistically significant (*p* < 0.05).

Supplementary Figure 3 Forest plots comparing the effects of **(A)** BAT vs. CT and **(B)** BAT vs. UAT on the MAL-quality of movement. 1 = bilateral functional training Test (BFTT); 2 = bilateral robot-assisted training (BRAT); 3 = bilateral arm training with rhythmic auditory cueing (BATRAC); 4 = mirror therapy (MT); BAT, bilateral arm training; CI, confidence interval; CT, conventional therapy; FE, fixed-effects; RE, random-effects; Std. Mean Diff., standardized mean difference; UAT, unilateral arm training; ^*^indicates statistically significant (*p* < 0.05).
