# Supplementary material for: Effectiveness and Success Factors of Bilateral Arm Training After Stroke: A Systematic Review and Meta-Analysis
Source: Front Aging Neurosci. 2022 Apr 25;14:875794. doi: 10.3389/fnagi.2022.875794 (PMC9082277; doi:10.3389/fnagi.2022.875794)
Supplement: Appendix 1 — Search strategies for meta-analysis. [file Presentation_1.PDF]

Appendix 1. Search Strategy

Using a combination of free-text terms and controlled vocabulary (MeSH), the following search strategy was used for MEDLINE via Ovid and was adapted for the other databases.

1. cerebrovascular disorders/ or exp basal ganglia cerebrovascular disease/ or exp brain ischemia/ or exp carotid artery diseases/ or exp cerebrovascular trauma/ or exp intracranial arterial diseases/ or exp intracranial arteriovenous malformations/ or exp "intracranial embolism and thrombosis"/ or exp intracranial hemorrhages/ or stroke/ or exp brain infarction/ or vasospasm, intracranial/ or vertebral artery dissection/
2. (stroke or poststroke or post-stroke or cerebrovasc$ or brain vasc$ or cerebral vasc$ or cva$ or apoplex$ or SAH).tw.
3. ((brain$ or cerebr$ or cerebell$ or intracran$ or intracerebral) adj5 (isch?emi$ or infarct$ or thrombo$ or emboli$ or occlus$)).tw.
4. ((brain$ or cerebr$ or cerebell$ or intracerebral or intracranial or subarachnoid) adj5 (haemorrhage$ or hemorrhage$ or haematoma$ or hematoma$ or bleed$)).tw.
5. hemiplegia/ or exp paresis/
6. (hemipleg$ or hemipar$ or paresis or paretic).tw.
7. 1 or 2 or 3 or 4 or 5 or 6
8. *cerebrovascular disorders/rh or exp *basal ganglia cerebrovascular disease/rh or exp *brain ischemia/rh or exp *carotid artery diseases/rh or exp *cerebrovascular trauma/rh or exp *intracranial arterial diseases/rh or exp *intracranial arteriovenous malformations/rh or exp *"Intracranial Embolism and Thrombosis"/rh or exp *intracranial hemorrhages/rh or *stroke/rh or exp *brain infarction/rh or *vasospasm, intracranial/rh or *vertebral artery dissection/rh
9. *hemiplegia/rh or exp *paresis/rh
10. 8 or 9
11. exp Upper Extremity/
12. (upper adj3 (limb$ or extremity)).tw.
13. (arm or shoulder or elbow or forearm or hand or wrist or finger or fingers).tw.
14. 11 or 12 or 13
15. rehabilitation/ or "recovery of function"/
16. Physical Therapy Modalities/ or Physical Therapy Specialty/
17. exercise movement techniques/ or exercise/ or exercise therapy/
19. "Task Performance and Analysis"/
20. occupational therapy/ or activities of daily living/
21. motor skills/ or "physical education and training"/
22. (rehabilitation or recovery of function or physiotherap$ or physical therap$ or exercise$ or movement$ or motor activit$ or occupational therap$ or activities of daily living or adl).tw.
23. ((bilateral or bimanual) adj5 (train$ or retrain$ or facilitat$ or function$ or

activit$)).tw.
24. ((mirror$ or coupled) adj5 movement$).tw.
25. 15 or 16 or 17 or 18 or 19 or 20 or 21 or 22 or 23 or 24
26. randomized controlled trial.pt.
27. controlled clinical trial.pt.
28. randomized.ab.
29. placebo.ab.
30. clinical trials as topic.sh.
31. randomly.ab.
32. trial.ti.
33. or/56-62
34. (animals not (humans and animals)).sh.
35. 63 not 64
36. 10 and 14 and 35
37. 7 and 14 and 25 and 35
38. 36 or 37
