# Supplementary figures and images for: Effectiveness and Success Factors of Bilateral Arm Training After Stroke: A Systematic Review and Meta-Analysis
Source: Front Aging Neurosci. 2022 Apr 25;14:875794. doi: 10.3389/fnagi.2022.875794 (PMC9082277; doi:10.3389/fnagi.2022.875794)

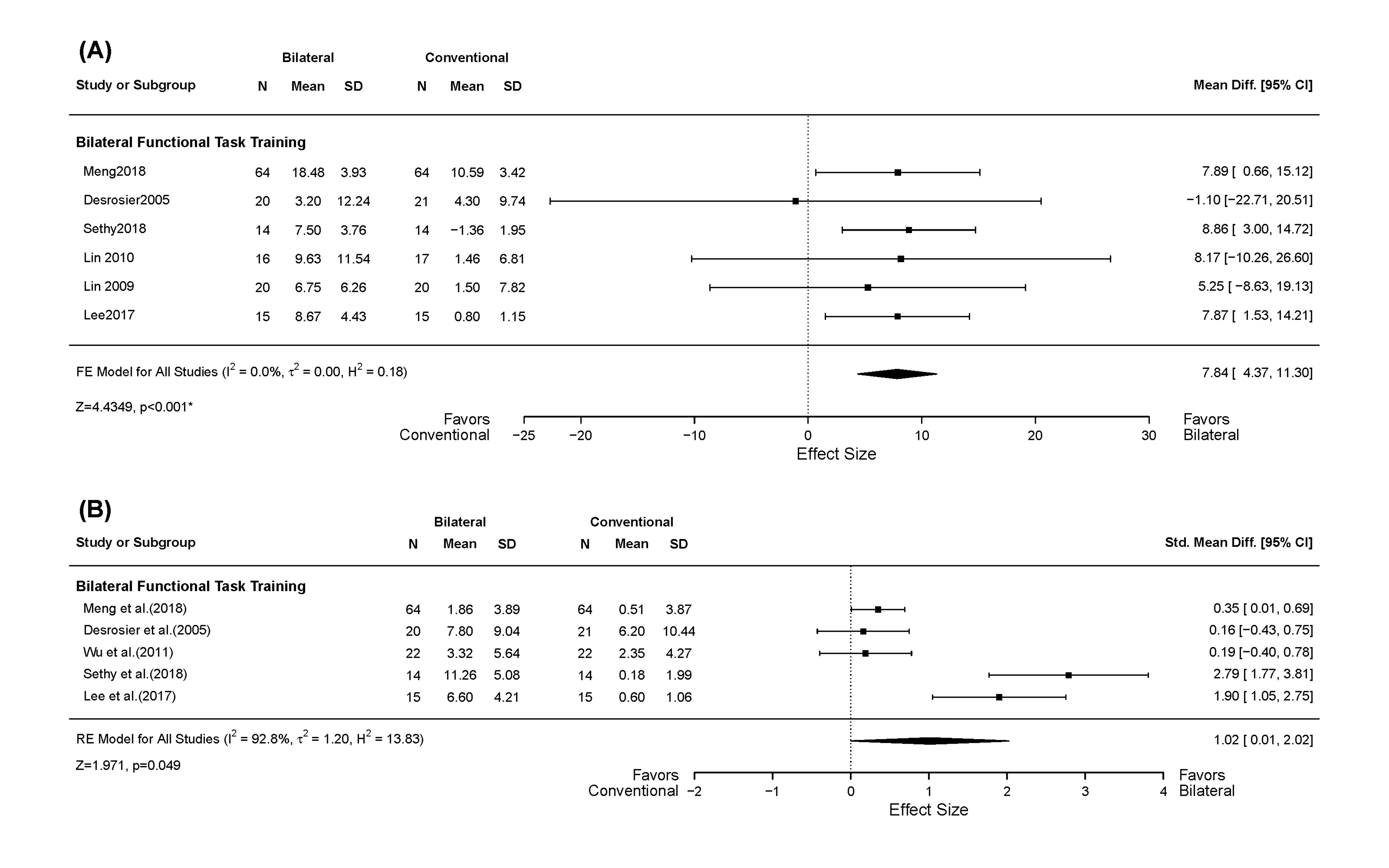

Supplement: Supplementary Figure 1 — The effects of bilateral functional task training (BFTT) on the upper extremity (A) motor impairments and (B) functional performance. BAT, bilateral arm training; CI, confidence interval; CT, conventional therapy; FE, fixed-effects; RE, random-effects; Std. Mean Diff., standardized mean difference. *indicates statistically significant (p < 0.05). [file Image_1.TIF]

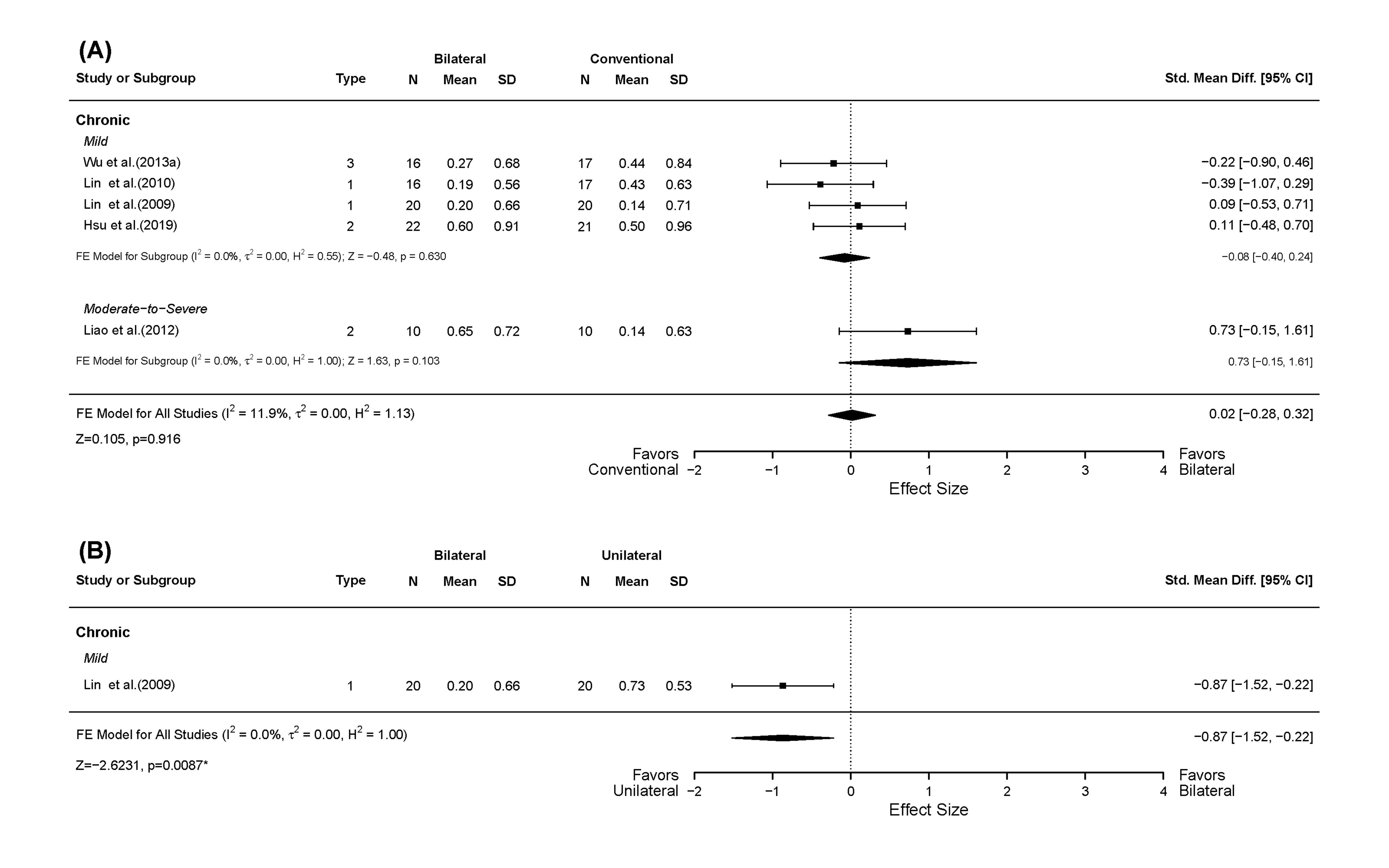

Supplement: Supplementary Figure 2 — Forest plots comparing the effects of (A) BAT vs. CT and (B) BAT vs. UAT on the MAL-amount of use. 1 = bilateral functional training Test (BFTT); 2 = bilateral robot-assisted training (BRAT); 3 = bilateral arm training with rhythmic auditory cueing (BATRAC); 4 = mirror therapy (MT); BAT, bilateral arm training; CI, confidence interval; CT, conventional therapy; FE, fixed-effects; RE, random-effects; Std. Mean Diff., standardized mean difference; UAT, unilateral arm training; *indicates statistically significant (p < 0.05). [file Image_2.TIF]

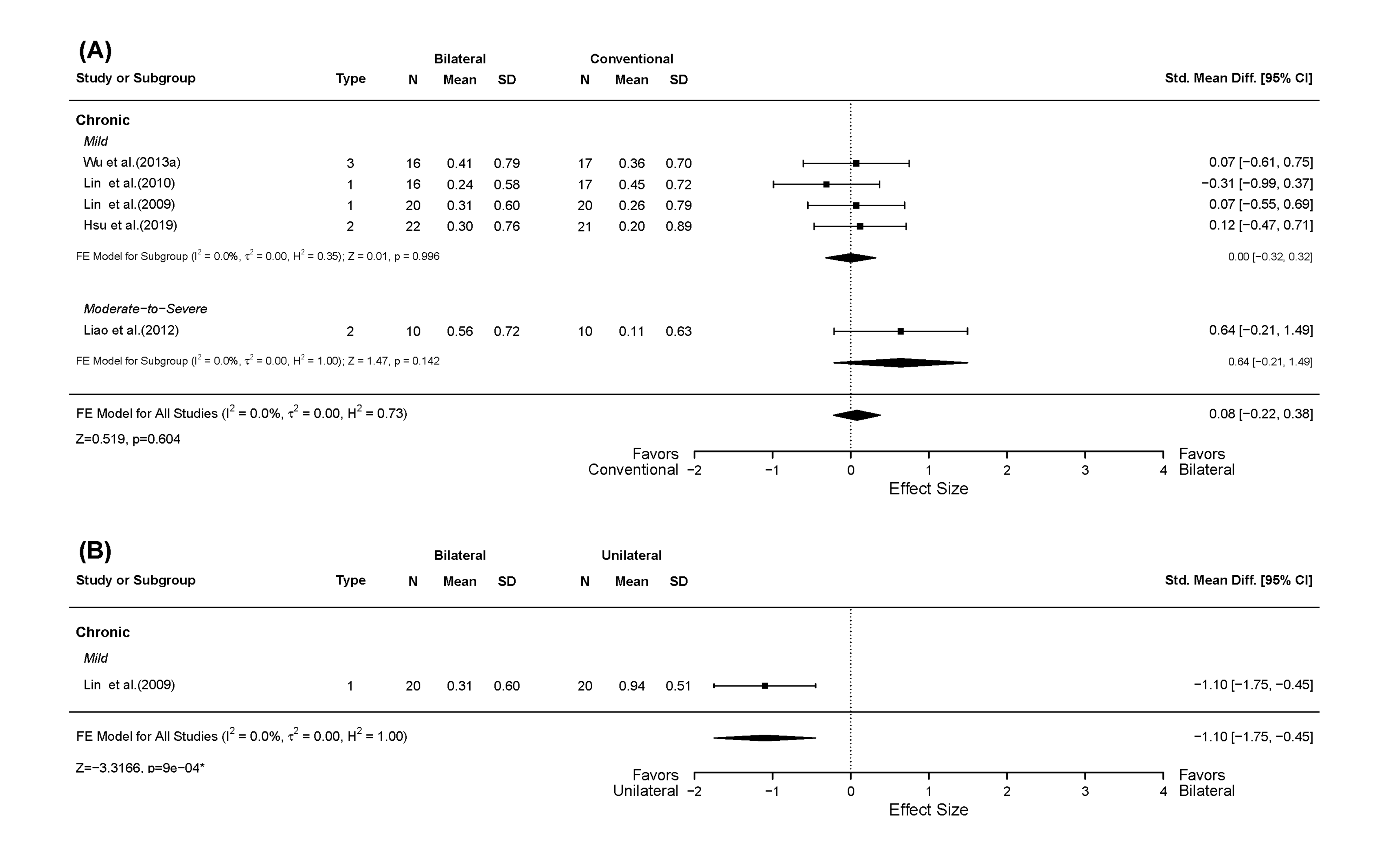

Supplement: Supplementary Figure 3 — Forest plots comparing the effects of (A) BAT vs. CT and (B) BAT vs. UAT on the MAL-quality of movement. 1 = bilateral functional training Test (BFTT); 2 = bilateral robot-assisted training (BRAT); 3 = bilateral arm training with rhythmic auditory cueing (BATRAC); 4 = mirror therapy (MT); BAT, bilateral arm training; CI, confidence interval; CT, conventional therapy; FE, fixed-effects; RE, random-effects; Std. Mean Diff., standardized mean difference; UAT, unilateral arm training; *indicates statistically significant (p < 0.05). [file Image_3.TIF]
